# Retrieval interference in reflexive processing: experimental evidence from Mandarin, and computational modeling

**DOI:** 10.3389/fpsyg.2015.00617

**Published:** 2015-05-27

**Authors:** Lena A. Jäger, Felix Engelmann, Shravan Vasishth

**Affiliations:** Department of Linguistics, University of PotsdamPotsdam, Germany

**Keywords:** Chinese reflexives, ACT-R, eye-tracking, interference, cue-based retrieval, computational modeling, ziji, content-addressable memory

## Abstract

We conducted two eye-tracking experiments investigating the processing of the Mandarin reflexive *ziji* in order to tease apart structurally constrained accounts from standard cue-based accounts of memory retrieval. In both experiments, we tested whether structurally inaccessible distractors that fulfill the animacy requirement of *ziji* influence processing times at the reflexive. In Experiment 1, we manipulated animacy of the antecedent and a structurally inaccessible distractor intervening between the antecedent and the reflexive. In conditions where the accessible antecedent mismatched the animacy cue, we found inhibitory interference whereas in antecedent-match conditions, no effect of the distractor was observed. In Experiment 2, we tested only antecedent-match configurations and manipulated locality of the reflexive-antecedent binding (Mandarin allows non-local binding). Participants were asked to hold three distractors (animate vs. inanimate nouns) in memory while reading the target sentence. We found slower reading times when animate distractors were held in memory (inhibitory interference). Moreover, we replicated the locality effect reported in previous studies. These results are incompatible with structure-based accounts. However, the cue-based ACT-R model of Lewis and Vasishth (2005) cannot explain the observed pattern either. We therefore extend the original ACT-R model and show how this model not only explains the data presented in this article, but is also able to account for previously unexplained patterns in the literature on reflexive processing.

## 1. Introduction

One major task the human parser has to accomplish is to syntactically link together two or more linguistic elements that are not adjacent to each other. For example, when a reflexive is being processed, it has to be somehow linked to its antecedent even if there is intervening material. Therefore, one central question in psycholinguistics is what mechanisms the human parser uses to identify and retrieve the previously processed part of a dependency. Theoretically, there are different options how this identification and retrieval of a linguistic constituent from working memory might be accomplished: different kinds of search mechanisms on the one hand (Sternberg, 1966, 1969) and cue-based, i.e., content-addressable, retrieval on the other hand (McElree and Dosher, 1989; Anderson and Lebiere, 1998; Anderson et al., 2004).^1^ In general, a search mechanism checks certain items in memory based on their location in order to find the target. Cue-based retrieval, in contrast, assumes that retrieval targets are content-addressable and can be accessed directly by the use of certain features as retrieval cues. Over the last decade, evidence favoring a content-addressable memory underlying human sentence processing has accumulated (McElree, 2000, 2003; McElree et al., 2003; Van Dyke and McElree, 2006; Martin and McElree, 2008).

In the case of English reflexives, retrieval cues used in a content-addressable memory might be non-structural cues like gender or number along with structural cues like local c-command. Note that a reflexive's binding domain varies between languages (Büring, 2005; Reuland, 2011). Whereas in English it can be approximated by the local clause, in Chinese the reflexive *ziji* can be bound across clause boundaries (non-local binding; for a brief overview of the syntactic properties of Chinese *ziji* see below). For the sake of simplicity, we will refer to the structural feature of *c-commanding the reflexive and being contained in its binding domain* briefly as the *c-command* feature.

However, within the framework of cue-based retrieval, it is still an open question which features the parser uses as retrieval cues. On the one hand, it has been proposed that all available cues are used for retrieval with equal weights being applied to all cues (Lewis and Vasishth, 2005). We will refer to this account as the *standard cue-based retrieval account*. On the other hand, Van Dyke (2007) and Van Dyke and McElree (2011) and others argue that syntactic cues (being in a certain tree-configurational position) have some kind of priority over non-syntactic cues. In particular, it has been proposed that for the processing of reflexive-antecedent dependencies, the set of features used for retrieving a reflexive's antecedent is limited to syntactic cues such as c-command within the reflexive's binding domain (Nicol and Swinney, 1989; Sturt, 2003; Xiang et al., 2009; Phillips et al., 2011; Dillon et al., 2013; Kush and Phillips, 2014). We will refer to this proposal as *structure-based account*.

If a structure-based retrieval is applied, a noun phrase that is in a structural position that disqualifies it from being the reflexive's antecedent should not have any effect on the processing of the reflexive-antecedent dependency, no matter whether it matches non-structural features of the reflexive such as gender or number. Thus, in a sentences like (1), the gender of *Jonathan* or *Jennifer* should not affect processing times of the reflexive since they do not c-command it and hence cannot syntactically bind the reflexive.

(1)  a. **Antecedent-match; distractor-match**
            The *surgeon* who treated *Jonathan* had pricked *himself* …
      b. **Antecedent-match; distractor-mismatch**
            The *surgeon* who treated *Jennifer* had pricked *himself* …
      c. **Antecedent-mismatch; distractor-match**
            The *surgeon* who treated *Jennifer* had pricked *herself* …
      d. **Antecedent-mismatch; distractor-mismatch**
            The *surgeon* who treated *Jonathan* had pricked *herself* …

The parsing architecture developed by Lewis and Vasishth (2005), which is based on Anderson et al. (2004)'s cognitive architecture ACT-R (Adaptive Control of Thought–Rational) assumes a cue-based retrieval mechanism without syntactic constraints. This model has been used to explain interference effects in sentence processing and in reflexives in particular (e.g., Dillon et al., 2013; Parker and Phillips, 2014; Patil, Vasishth, and Lewis, “Retrieval interference in syntactic processing: The case of reflexive binding in English,” unpublished manuscript). According to the ACT-R model, both latency and probability of retrieving a certain target item are determined by (i) the quality of the match between retrieval cues and target features and (ii) similarity-based mutual inhibition between the target and other matching items. Retrieval speed and probability increase with the number of cues matching the target. If, however, certain cues match the features of multiple memory items, similarity-based interference leads to a higher retrieval latency, i.e., inhibitory interference effects. The latter is the case in (1a) as compared to (1b), because in (1a) both the target *surgeon* and the distractor *Jonathan* share the feature +*masculine*. In the antecedent-mismatch conditions (1c) vs. (1d), in contrast, the target *surgeon* and the cue-matching distractor *Jennifer* in (1c) do not share the feature +*feminine*, hence, no similarity-based interference arises. Consequently, no inhibition is predicted in (1c) vs. (1d). On the contrary, because both target and distractor only partially match the retrieval cues in (1c), they are equally likely to be retrieved. Compared to (1d), this predicts a higher proportion of incorrect retrievals and a lower average retrieval latency, which is usually referred to as *facilitatory interference* or *intrusion*.

In sum, a major prediction that distinguishes standard cue-based retrieval from models assuming a limitation of the retrieval cues to structural features is that the former entails interference effects from non-target items that match (some of) the cues used for retrieval.^2^

In order to tease apart structure-based from standard cue-based retrieval, interference effects from feature-matching but syntactically illicit antecedents in the processing of reflexive-antecedent dependencies have drawn considerable attention in recent years. Several studies used a feature-match/mismatch design, where a non-syntactic feature (e.g., gender or number) was manipulated at the antecedent and at a structurally inaccessible distractor (see Example 1 for typical sentence material). In Table 1, we provide an overview of the studies examining interference effects in reflexives (including reflexives inside a prepositional phrase and possessive reflexives) and reciprocals using a feature-match/mismatch design. Studies on the processing of reflexives in so-called picture noun phrases have not been included in our review since their binding properties differ from other reflexives (Büring, 2005; Reuland, 2011). Moreover, experiments investigating specific populations such as children or L2 learners are not considered in the review. Table 1 summarizes whether or not inhibitory (i.e., a slowdown due to the presence of a cue-matching inaccessible distractor) or facilitatory (i.e., a speed-up due to the presence of a cue-matching inaccessible distractor) interference was observed in (i) conditions with an accessible antecedent that matched the feature under examination and (ii) conditions with an accessible antecedent that mismatched the feature under examination (i.e., sentences that are either ungrammatical or at least violating the stereotypical gender of the accessible antecedent). Some studies manipulated other factors in addition to the feature-match/mismatch manipulation. In these cases, we split the respective experiments into two entries in Table 1, with one entry for each level of the additional factor. In particular, for Felser et al. (2009), who manipulated feature type (gender vs. c-command) as additional within-participants factor and language proficiency (native speaker vs. L2 learner) as between-participants factor, one row in Table 1 refers to the manipulation of the c-command feature in native speakers and another row refers to the gender manipulation in native speakers. The results of the non-native group are not included in the table because this review concerns adult native speaker populations. For Chen et al. (2012), who manipulated whether the Chinese reflexive *ziji* was locally or non-locally bound, one row in Table 1 refers to the interference effect observed in conditions with a local antecedent and a second row refers to the conditions with a non-local antecedent. Similarly, in the case of King et al. (2012), who manipulated whether the reflexive directly followed the verb or a preposition intervened, one table entry refers to the former configuration (labeled as *adjacent*) and another entry refers to the latter configuration (labeled as *non-adjacent*). In the review of Clackson et al. (2011), who primarily investigated the processing of reflexives in children, we only report the results of the adult control group. For the reviewed experiments, we report effects observed at the region containing the reflexive (labeled as *crit*) and the following regions (labeled as *crit* + *x*). Although the size of the interest areas in terms of number of words contained in one region differs between studies, which reduces the comparability of the time course of the observed effects to a certain extent, we keep the sectioning of the interest areas as in the respective publication.

**Table 1:** **Overview of interference effects at reflexives categorized for feature-match/mismatch of the accessible antecedent and direction of the effect (inhibitory vs. facilitatory interference)**.

	**Publication**	**Lang**.	**Method**	**Cue**	**Depend**.	**Interf**.	**Distractor**	**Effect in antecedent-match**		**Effect in antecedent-mismatch**	
					**type**	**type**	**position**	***Interference***	***AOI, Measure***	***Interference***	***AOI, Measure***
1	Xiang et al. '09	EN	EEG	gend	refl	retro	subj.	—		(inhib)	
2	Badecker and Straub '02 Exp5	EN	SPR	gend	refl	pro	genitive	*n.s.*		—	
3	Badecker and Straub '02 Exp6	EN	SPR	gend	refl	pro	prep.obj.	*n.s.*		—	
4	Chen et al. '12 local	CN	SPR	anim	poss.refl.	retro	subj.	*n.s.*		—	
5	Clackson et al. '11 Exp2 adults	EN	VW	gend	prep.refl.	pro	subj.(topic)	*n.s.*		—	
6	Clifton et al. '99 Exp1	EN	SPR	num	refl	retro	prep.obj.	*n.s.*		—	
7	Clifton et al. '99 Exp2	EN	SPR	gend,num	refl	retro	prep.obj.	*n.s.*		—	
8	Clifton et al. '99 Exp3	EN	SPR	gend	refl	pro	subj	*n.s.*		—	
9	Felser et al. '09 natives, gend	EN	ET	gend	refl	pro	subj.(topic)	*n.s.*		—	
10	Nicol and Swinney '89	EN	Primg	gend	refl	pro	subj.,obj.	*n.s.*		—	
11	Dillon et al. '13	EN	ET	num	refl	retro	obj.	*n.s.*		*n.s.*	
12	King et al. '12 adjacent	EN	ET	gend	refl	retro	obj.	*n.s.*		*n.s.*	
13	Sturt '03 Exp2	EN	ET	gend	refl	retro	obj.(topic)	*n.s.*		*n.s.*	
14	Cunnings and Felser '13 Exp1	EN	ET	gend	refl	pro	subj.(topic)	*n.s.*		(facil)	crit+2, FPRT
15	King et al. '12 non-adjacent	EN	ET	gend	prep.refl.	retro	prep.obj.	*n.s.*		facil	crit, FPRT
16	Parker and Phillips '14	EN	ET	nu/ge/an	refl	pro	subj.	*n.s.*		facil	crit, TFT
17	Cunnings and Sturt '14 Exp1	EN	ET	gend	refl	pro	subj.(topic)	*n.s.*		(inhib)	crit+1, FPRT
18	Kush and Phillips '14	HI	SPR	num	recipr	retro	prep.obj.	*n.s.*		(inhib)	crit+2
19	**This study Exp1**	CN	ET	anim	refl	retro	subj.	*n.s.*		inhib	crit, FFD / FPRT / RBRT / RPD
20	Cunnings and Felser '13 Exp2	EN	ET	gend	refl	retro	subj.(topic)	facil *lWM*	crit, FFD / FPRT	(inhib *lWM*)	crit, FFD
21	Sturt '03 Exp1	EN	ET	gend	refl	pro	subj.(topic)	facil	crit+2, RRT	*n.s.*	
22	**This study Exp2**	CN	ET	anim	refl	pro	memory	inhib	crit, FPRT / RBRT / RPD / TFT	—	
23	Badecker and Straub '02 Exp3	EN	SPR	gend	refl	pro	subj.	inhib	crit+1	—	
24	Badecker and Straub '02 Exp4	EN	SPR	num	recipr	pro	subj.	inhib	crit+1–crit+4	—	
25	Chen et al. '12 non-local	CN	SPR	anim	poss.refl.	retro	subj.	inhib	crit+1	—	
26	Clackson and Heyer '14	EN	VW	gend	prep.refl.	pro	subj.(topic)	inhib	gaze shift	—	
27	Felser et al. 09 natives, c-com	EN	ET	c-com	refl	pro	subj.(topic)	inhib	crit, RPD	—	
28	Patil et al. unpublished	EN	ET	gend	refl	retro	subj.	inhib	crit, FPRP / (reg.-cont. FFD)	(facil)	crit, regr.-cont. FFD

Experiments are sorted by effect pattern (effect in antecedent-match first, effect in antecedent-mismatch second). Marginal effects are in parentheses. The experiments are classified by language (English EN, Mandarin Chinese CN, Hindi HI), experimental method (self-paced reading SPR, eye-tracking while reading ET, visual world eye-tracking VW, event-related potentials ERP, cross-modal priming Primg), retrieval cues that are examined (gender, number, animacy, c-command), dependency type (reflexive, prepositional reflexive, possessive reflexive, reciprocal), interference type (proactive vs. retroactive), and by syntactic position of the distractor (subject, object, genitive attribute, sentence external memory load, and discourse topicality in parenthesis). For reading studies, the interest area (AOI) labeled *crit* refers to the region containing the reflexive/reciprocal. For eye-tracking experiments, Measure indicates the dependent variable in which the effect was observed (first-fixation duration FFD, first-pass reading time FPRT, total-fixation time TFT, regression-path duration RPD, right-bounded reading time RBRT, re-reading time RRT, first-pass regression probability FPRP, first-fixation duration in regression-contingent trials reg.-cont. FFD). Effects in parentheses are only marginally significant. The entry “—” means that the respective conditions were not tested in the experiment while “*n.s.*” means that no significant effect was observed. *lWM* refers to participants with low working memory capacity.

In accessible antecedent-match conditions, previous studies found inhibitory interference in six cases (Badecker and Straub, 2002, Experiments 1 and 2; Felser et al., 2009, c-command manipulation in native speakers; Chen et al., 2012, non-local reflexives; Clackson and Heyer, 2014; Patil, Vasishth, and Lewis, “Retrieval interference in syntactic processing: The case of reflexive binding in English,” unpublished manuscript). Statistically significant facilitatory interference in antecedent-match conditions was found in two experiments (Sturt, 2003, Experiment 1; Cunnings and Felser, 2013, Experiment 2). However, Sturt found the effect only in re-reading time two words after the reflexive and this effect could not be replicated by Cunnings and Sturt (2014), who used similar stimuli. Cunnings and Felser found the effect for readers with low working memory span (*lWM*), but not for high-span readers. In the majority of the experiments, in contrast, no interference effect was observed in antecedent-match conditions (Nicol and Swinney, 1989; Clifton et al., 1999; Badecker and Straub, 2002, Experiments 5 and 6; Sturt, 2003, Experiment 2; Felser et al., 2009, gender manipulation in native speakers; Clackson et al., 2011, adult control group of Experiment 2; Chen et al., 2012, conditions with local reflexive binding; King et al., 2012, adjacent conditions; Cunnings and Felser, 2013, Experiment 1; Dillon et al., 2013; Kush and Phillips, 2014; Cunnings and Sturt, 2014, Experiment 1; Parker and Phillips, 2014).^3^

For conditions with a feature-mismatching accessible antecedent, two studies report significant effects of facilitatory interference (King et al., 2012; Parker and Phillips, 2014) and two studies report a marginal facilitatory effect (Cunnings and Felser, 2013, Experiment 1; Patil, Vasishth, and Lewis, “Retrieval interference in syntactic processing: The case of reflexive binding in English,” unpublished manuscript)—however, the latter effect was only found in a *post-hoc* analysis of regression-contingent first-fixation durations, and thus might be spurious. Marginal effects of inhibitory interference have been reported for participants with low working memory span (Cunnings and Felser, 2013, Experiment 2), in the processing of reciprocals (Kush and Phillips, 2014), and in Experiment 1 of Cunnings and Sturt (2014). The latter only report a marginal main effect of the distractor, but their reported means suggest that the effect was driven by the antecedent-mismatch conditions. This does, however, not seem very reliable because they used similar stimuli as Sturt (2003), Experiment 1, who, in contrast, had not found an effect in antecedent-mismatch conditions but a facilitation in antecedent-match conditions. A general pattern is that interference effects in antecedent-match conditions are less frequently observed than effects in antecedent-mismatch conditions.

To summarize, the literature on reflexive interference contains a mixture of results, not favoring one particular of the retrieval models in question. Studies showing a general absence of interference support structure-based accounts (Nicol and Swinney, 1989; Sturt, 2003; Xiang et al., 2009; Phillips et al., 2011; Dillon, 2011; Dillon et al., 2013; Kush and Phillips, 2014). On the other hand, observations of significant interference effects have been interpreted as evidence against purely structure-based retrieval (Badecker and Straub, 2002; Chen et al., 2012; Clackson and Heyer, 2014; Parker and Phillips, 2014). Crucially, however, taking into account the direction of the effects, there are patterns that cannot be explained by either account without employing additional assumptions: The cue-based retrieval account as implemented by Lewis and Vasishth (2005) and employed by Dillon (2011), Dillon et al. (2013), Kush and Phillips (2014), Parker and Phillips (2014) and Patil, Vasishth, and Lewis, “Retrieval interference in syntactic processing: The case of reflexive binding in English” (unpublished manuscript) is unable to explain facilitatory interference in antecedent-match conditions or inhibitory interference in antecedent-mismatch conditions.

The present article (i) provides further experimental evidence relating to the current debate about the use of non-structural retrieval cues and (ii) proposes two extensions to the standard cue-based retrieval architecture in order to account for the seemingly contradictory patterns of experimental results observed across studies.

We first present two eye-tracking experiments examining interference effects in the processing of the Mandarin Chinese reflexive *ziji*. There is a wide range of competing syntactic or pragmatic approaches of how to analyze *ziji* (for formal accounts see Yang, 1983; Manzini and Wexler, 1987; Pica, 1987; Kang, 1988; Tang, 1989; Huang and Tang, 1989, 1991; Cole et al., 1990, 1993; Cole and Sung, 1994; Cole and Wang, 1996; for pragmatic and non-uniform accounts see Huang et al., 1984; Yu, 1992, 1996; Xue et al., 1994; Pan, 1997; Pollard and Xue, 1998; Huang and Liu, 2001; Liu, 2010). We will restrict the following summary of the syntactic behavior of *ziji* to its properties that are relevant for the present experimental design. In contrast to English reflexives, *ziji* does not have any gender or number marking, but requires its antecedent to be animate (Tang, 1989).^4^ Thus, animacy might be used as a non-structural cue to retrieve *ziji*'s antecedent. Similar to reflexives of many other languages including English, *ziji* needs to be c-commanded by its antecedent.^5^ Moreover, the antecedent is required to be a subject (Huang, 1984). In contrast to English, the antecedent does not have to be contained in the local clause of the reflexive, but can also be contained in a superordinate clause (non-local binding). The processing of locally vs. non-locally bound *ziji* has been investigated by Gao et al. (2005), Liu (2009), Li and Zhou (2010), Dillon (2011), Chen et al. (2012), and Dillon et al. (2014).

The present experiments examine whether animate nouns that are in a structurally inaccessible position (i.e., not c-commanding the reflexive) induce interference effects on the processing of *ziji*. So far, the literature on interference effects in reflexives has focused on morphologically marked phi-features (gender, number). Thus, the examination of animacy in the processing of Mandarin *ziji* does not only add cross-linguistic evidence to the debate that, so far, has been centered on English, but also extends the range of investigated retrieval cues to a purely semantic feature.

Both experiments have relatively large sample sizes in order to increase statistical power. Given that the prediction of the structure-based account is that no effect should be seen (i.e., a null result), it is particularly important to conduct high power studies.

## 2. Experiment 1

In Experiment 1, we tested whether locally bound *ziji* is subject to interference from a structurally inaccessible distractor that fulfills the animacy requirement of *ziji*. In a 2 × 2 design we manipulated animacy of the structurally accessible antecedent (henceforth labeled as *antecedent-match* vs. *antecedent-mismatch*) and of a structurally inaccessible distractor noun that intervened between the accessible antecedent and the reflexive (henceforth labeled as *distractor-match* vs. *distractor-mismatch*). This design extends the study reported by Chen et al. (2012), who were the first to test interference effects in Mandarin *ziji*, in several respects. In contrast to Chen and colleagues, in the present experiment, *ziji* was in object position rather than being a possessive modifier and we included antecedent-mismatch conditions which Chen et al. did not test. Moreover, we used the more time-sensitive eye-tracking method rather than self-paced reading.

The ACT-R model as implemented by Lewis and Vasishth (2005) predicts an inhibitory interference effect in antecedent-match conditions and a facilitatory interference effect in antecedent-mismatch conditions at the reflexive. The structure-based account (Nicol and Swinney, 1989; Sturt, 2003; Phillips et al., 2011; Dillon, 2011; Dillon et al., 2013; Kush and Phillips, 2014), in contrast, predicts the absence of an interference effect in both antecedent-match and antecedent-mismatch conditions. Moreover, the Lewis and Vasishth ACT-R model predicts incorrect retrievals of the animate distractor (misretrievals) in both antecedent-match and antecedent-mismatch conditions, but the proportion of misretrievals is predicted to be higher in antecedent-mismatch conditions. The structure-based account predicts no misretrievals of the animate inaccessible distractor.

### 2.1. Materials and method

#### 2.1.1. Materials

We tested 48 experimental sentences which contained an either animate (antecedent-match) or inanimate (antecedent-mismatch) accessible antecedent in subject position (*yundongyuan* “athlete” vs. *pihuating* “kayak” in 2) and the reflexive as direct object. Due to the animacy requirement of *ziji*, the conditions with an inanimate accessible antecedent were ungrammatical. Between the main clause subject and the main clause verb, an adverbial clause intervened that contained an either animate (distractor-match) or inanimate (distractor-mismatch) inaccessible distractor (*lingdui* “team leader” vs. *meiti* “media” in 2). This distractor was also a subject, but did not c-command the reflexive and was therefore not a legal antecedent. The reflexive was followed by a frequency phrase or a durational phrase consisting of four characters, which was analyzed as a spillover region.

**Table:** 

(2)	**Animate/Inanimate antecedent; Animate/Inanimate distractor**								
	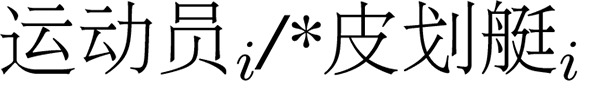	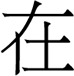	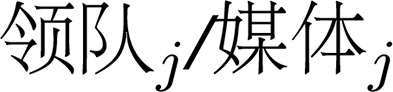	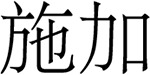	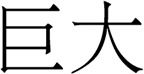	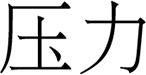	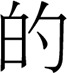	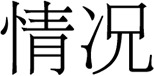	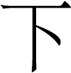
	**Yundongyuan**_*i*_/^*^Pihuating_*i*_ [pp	zai	**lingdui**_*j*_/**meiti**_*j*_	shijia	juda	yali	de	qingkuang	xia]
	*athlete*/*kayak*	*when*	*team.leader/media*	*excert*	*great*	*pressure*	mod	*circumstance*	*under*
	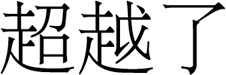 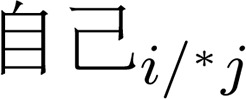 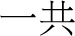	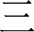	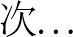						
	chaoyue-le **ziji**_*i*/^*^j_ yigong	san	ci…						
	*outperform*-asp *self in total*	*three*	*times*…						
	*When the team leader/media excerted great pressure, the athlete/kayak outperformed himself/itself three times in total*…								

The experimental items were complemented with 72 filler sentences (48 grammatical, 24 ungrammatical) with varying syntactic structures including sentences containing the bare reflexive *ziji* as well as the bi-morphemic reflexive *ta-ziji* and pronouns in different syntactic positions.

Each sentence was followed by a multiple choice comprehension question that probed for the correct retrieval of the antecedent. Participants could choose between the antecedent, the distractor, an unrelated noun taken from a previous trial and the option “I am not sure.” This design allowed us to examine not only whether the antecedent was retrieved correctly, but also to assess the proportion of misretrievals of the distractor. To ensure that participants also fully parsed the intervening adverbial clause containing the distractor, a second multiple-choice question targeted the adverbial clause. The same options were provided as in the first question. The questions following the filler sentences targeted various syntactic positions in the sentence.

*Pretest*. Since the exact binding properties of *ziji* are still subject to discussion in the syntactic literature, we conducted a paper-based questionnaire study to test our assumption that the main clause subject in the experimental items binds the reflexive. Forty native speakers of Mandarin recruited at Beijing Normal University participated in this study against payment of 25 RMB (approximately 3 EUR). None of them would participate in either of the eye-tracking experiments. Participants were presented with the antecedent-match conditions of the experimental items together with 90 filler sentences containing *ziji* in various syntactic positions and were instructed to circle the word in the given sentence *ziji* referred to or to explicitly write down the referent in case of an unbound interpretation of *ziji*.

*Results*. In 97.2% of all trials, participants selected the main clause subject as antecedent for the reflexive (97.0% and 97.3% when the distractor was animate or inanimate, respectively). This shows that in the experimental materials, Mandarin speakers indeed choose the main clause subject as antecedent for the reflexive.

#### 2.1.2. Participants and procedure

The experiment was conducted in the eye-tracking lab of the State Key Laboratory of Cognitive Neuroscience and Learning at Beijing Normal University. One hundred fifty students from different universities located in Beijing participated in the experiment against payment of 40 RMB (approximately 5 EUR). All participants were native speakers of Mandarin and had normal or corrected to normal vision.

Eye movements (right eye monocular) were recorded using an SR Research Eyelink 1000 eyetracker at a sampling rate of 1000 Hz. Participants' head was stabilized using a forehead- and chin-rest. The screen-to-eye-distance was 82 cm, the camera-to-eye-distance 75 cm. Stimuli were presented in Simplified Chinese characters (font type SimSun, black font, font size 25) on a 22 inch monitor with light gray background using SR Research Experiment Builder software. Re-calibrations were performed between trials if necessary. Each experimental session began with 6 practice trials in which feedback to the comprehension questions was provided. In the experimental trials, no feedback was given. Short breaks were given according to the participants' individual needs. The sentences were presented according to a standard Latin Square. Items were pseudo-randomized such that at least one filler sentence intervened between two experimental sentences. Each sentence was followed by two multiple choice comprehension questions as described above.

### 2.2. Results

All statistical analyses were carried out in R using linear mixed effects models provided by the lme4 package version 1.0-6 (Bates et al., 2014). Binary dependent variables were analyzed using a logistic link function. For both, the analysis of response accuracies and eye movements, two sets of contrasts were applied. We first ran a model testing for a main effect of antecedent (animate antecedents coded as +0.5; inanimate antecedents coded as −0.5), a main effect of interference (animate distractors coded as +0.5; inanimate distractors coded as −0.5) and the interaction between the two main effects. Second, we applied nested contrasts testing for an interference effect within antecedent-match and antecedent-mismatch conditions separately. All models were fit with a full variance-covariance matrix for participants and items (Gelman and Hill, 2007); in case the model failed to converge or the variance-covariance matrix was degenerate, random slopes for items or participants were removed.

#### 2.2.1. Comprehension questions

Comprehension questions targeting the reflexive-antecedent dependency were analyzed. We analyzed response accuracies and the proportion of incorrect selection of the inaccessible distractor. An overview of participants' answers is provided in Table 2. In the statistical analysis of response accuracies, only the main effect of antecedent reached marginal significance (estimate = 0.34, *SE* = 0.18, *z* = 1.84, *p* = 0.07). The antecedent (i.e., the correct option) was chosen more often in antecedent-match conditions. This effect was expected since in the antecedent-mismatch conditions, no fully grammatically correct answer to the comprehension question was available (the antecedent was coded as “correct” answer, but the option “not sure” was provided as one response option in order to account for the ungrammaticality of the sentence). The analysis of the proportions of incorrect selection of the distractor revealed a main effect of antecedent: participants chose the distractor more often in antecedent-mismatch conditions than in antecedent-match conditions (estimate = −0.45, *SE* = 0.18, *z* = −2.48, *p* < 0.05). However, the size of this main effect was very small. We will therefore not base any conclusions on this effect. Moreover, the interaction between antecedent and distractor was significant (estimate = 0.56, *SE* = 0.15, *z* = 3.61, *p* < 0.001). Pairwise comparisons revealed that, within antecedent-match conditions, the distractor was chosen more often erroneously as answer to the comprehension question in case the distractor was animate (estimate = 0.83, *SE* = 0.31, *z* = 2.70, *p* < 0.01). But, as can be seen from Table 2, the animate distractor did not cause a decrease in selection probability of the antecedent but rather attracted selections from the unrelated noun. In antecedent-mismatch conditions, no interference effect was observed.

**Table 2:** **Experiment 1: Chosen answer to the comprehension question by condition in percentages**.

***Antecedent***	***Distractor***	**Chosen answer**			
		**Antecedent**	**Distractor**	**Unrelated**	**“Not sure”**
*match*	*match*	82.3	5.1	0.9	11.7
	*mismatch*	81.6	3.6	2.4	12.4
*mismatch*	*match*	75.9	4.8	1.1	18.2
	*mismatch*	75.7	4.9	0.8	18.5

#### 2.2.2. Eye movements

Eye movements were analyzed at the reflexive, the pre-critical region (verb) and the spillover material consisting of the frequency/durational phrase (post-critical). In order to provide a comprehensive picture of our data, and to make our results comparable to other studies we report the whole range of eye-tracking measures common in psycholinguistic research, although some of these measures are correlated by definition. As first-pass measures, we report first-fixation duration (FFD), i.e., the duration of the first fixation in first-pass reading, and first-pass reading time (FPRT, also called gaze duration), i.e., the sum of all first-pass fixations on a word before leaving it. As regression-related measures, we report regression-path duration (RPD, also called go-past time), i.e., the sum of all fixation durations starting from the first first-pass fixation on a word including regressive fixations to previous material until a region to the right of this word is fixated, right-bounded reading time (RBRT), i.e., the sum of all fixations on a word before another region to the right of this region is fixated, and first-pass regression probability (FPRP), i.e., the proportion first-pass regressions initiated from a word. As a later-pass measure, we analyzed re-reading time (RRT), i.e., the sum of all fixations on a word that are not contained in FPRT. In addition, we analyzed total-fixation time (TFT), which is defined as the sum of FPRT and RRT. In order to achieve close to normally distributed model residuals, we log-transformed reading times (Box and Cox, 1964) and excluded all trials in which the respective continuous dependent variable was zero. First-fixation probability of the pre-critical region, the reflexive and the spillover region was 90, 62, and 87%, respectively. Re-readings occurred in 60, 33, and 45% of the trials at pre-critical region, the reflexive and the spillover region, respectively. In all models, centered log-frequencies of the antecedent and the distractor taken from the SUBLETEX-CH database (Cai and Brysbaert, 2010) were included as covariates because items had not been matched for frequencies of the antecedents and distractors. Mean raw reading times with standard errors for the pre-critical, critical and post-critical regions are provided in Table 3. The results of the statistical analyses of participants' eye movements are summarized in Tables 4, 5.

**Table 3:** **Experiment 1: Means and standard errors of raw first-fixation duration, first-pass reading time, right-bounded reading time, regression-path duration, total fixation time, re-reading time in ms, and first-pass regression probability in percentages at the pre-critical region, the reflexive and the post-critical region**.

	**Pre-critical**				**Reflexive**				**Post-critical**			
***Antecedent***	***Match***		***Mismatch***		***Match***		***Mismatch***		***Match***		***Mismatch***	
***Distractor***	***Match***	***Mism***.	***Match***	***Mism***.	***Match***	***Mism***.	***Match***	***Mism***.	***Match***	***Mism***.	***Match***	***Mism***.
FFD	279 (3)	277 (3)	285 (3)	279 (3)	258 (3)	259 (3)	264 (4)	251 (3)	270 (4)	274 (4)	274 (3)	268 (4)
FPRT	366 (6)	370 (6)	386 (6)	375 (6)	269 (4)	270 (4)	282 (5)	263 (4)	376 (8)	370 (8)	384 (7)	364 (7)
RBRT	397 (6)	407 (7)	425 (7)	413 (7)	286 (4)	284 (4)	302 (5)	284 (4)	436 (9)	430 (9)	448 (9)	432 (9)
RPD	484 (13)	508 (14)	537 (15)	533 (15)	430 (16)	410 (15)	484 (18)	494 (25)	688 (25)	662 (23)	759 (27)	755 (30)
FPRP	13 (1)	14 (1)	14 (1)	16 (1)	17 (1)	15 (1)	19 (1)	17 (1)	24 (1)	24 (1)	26 (1)	26 (1)
TFT	725 (14)	696 (14)	761 (15)	716 (14)	439 (10)	428 (9)	455 (10)	433 (9)	628 (14)	614 (15)	628 (15)	605 (13)
RRT	577 (17)	537 (16)	604 (17)	565 (16)	418 (15)	396 (14)	411 (14)	397 (13)	507 (18)	503 (20)	509 (21)	493 (17)

In the calculation of standard errors of continuous dependent variables, between-participants variance has been removed using the Cousineau (2005) normalization with Morey (2008)'s correction. For continuous variables, trials with a 0 as value of the respective variable have been excluded.

**Table 4:** **Experiment 1: Main effect of antecedent, main effect of interference and their interaction at the pre-critical (*****ziji*** **− 1), critical (*****ziji*****), and post-critical (*****ziji*** **+ 1) regions for the dependent variables (DVs) first-fixation duration, first-pass reading time, right-bounded reading time, regression-path duration, first-pass regression probability, total fixation time, and re-reading time**.

**DV**	**Comparison**	**Pre-critical**			**Reflexive**			**Post-critical**		
		***Coef***	***SE***	***t or z***	***Coef***	***SE***	***t or z***	***Coef***	***SE***	***t or z***
FFD	Antecedent	−0.02	0.01	−1.72	0.00	0.01	−0.27	0.00	0.01	−0.10
	Interference	0.01	0.01	0.84	0.02	0.01	1.42	0.01	0.01	0.45
	Ant × Int	−0.01	0.01	−0.61	**−0.02**	**0.01**	−2.06^*^	−0.02	0.01	−1.77
FPRT	Antecedent	−0.03	0.01	−1.93	−0.02	0.01	−1.02	−0.02	0.02	−0.96
	Interference	0.01	0.01	0.71	0.03	0.02	1.94	**0.04**	**0.01**	2.69^*^
	Ant × Int	−0.02	0.01	−1.71	**−0.04**	**0.01**	−2.92^*^	−0.02	0.01	−1.24
RBRT	Antecedent	**−0.04**	**0.01**	−2.78^*^	**−0.04**	**0.02**	−2.23^*^	**−0.04**	**0.02**	−2.65^*^
	Interference	0.00	0.01	−0.20	0.02	0.02	1.59	**0.03**	**0.01**	2.12^*^
	Ant × Int	**−0.03**	**0.01**	−2.02^*^	**−0.03**	**0.01**	−2.14^*^	−0.01	0.01	−0.56
RPD	Antecedent	**−0.06**	**0.02**	−2.57^*^	**−0.09**	**0.02**	−3.98^*^	**−0.09**	**0.03**	−3.14^*^
	Interference	−0.02	0.02	−1.11	**0.04**	**0.02**	2.25^*^	0.03	0.02	1.11
	Ant × Int	−0.02	0.02	−1.30	−0.01	0.02	−0.73	−0.01	0.02	−0.32
FPRP	Antecedent	−0.08	0.10	−0.79	**−0.18**	**0.08**	−2.17^*^	−0.12	0.09	−1.41
	Interference	−0.14	0.10	−1.46	**0.16**	**0.07**	2.14^*^	0.03	0.07	0.42
	Ant × Int	0.03	0.09	0.35	0.01	0.07	0.19	0.03	0.06	0.43
TFT	Antecedent	**−0.04**	**0.02**	−2.20^*^	**−0.04**	**0.02**	−2.29^*^	−0.01	0.02	−0.64
	Interference	**0.04**	**0.01**	2.76^*^	0.02	0.02	1.48	0.03	0.02	1.93
	Ant × Int	−0.01	0.01	−0.83	−0.02	0.02	−1.12	0.01	0.02	0.57
RRT	Antecedent	**−0.06**	**0.03**	−2.22^*^	−0.05	0.03	−1.47	−0.02	0.03	−0.68
	Interference	0.04	0.02	1.74	0.02	0.03	0.87	−0.01	0.03	−0.46
	Ant × Int	0.02	0.02	0.72	−0.01	0.03	−0.42	0.03	0.03	1.06

Statistically significant (α = *0.05*) effects are marked with an asterisk and highlighted in bold.

**Table 5:** **Experiment 1: Pairwise comparisons of animacy of the distractor (interference) nested within animate/inanimate antecedent (antecedent match/mismatch) at the pre-critical (*****ziji*** **− 1), critical (*****ziji*****), and post-critical (*****ziji*** **+ 1) regions for the dependent variables (DVs) first-fixation duration, first-pass reading time, right-bounded reading time, regression-path duration, first-pass regression probability, total fixation time, and re-reading time**.

**DV**	**Comparison**	**Pre-critical**			**Reflexive**			**Post-critical**		
		***Coef***	***SE***	***t or z***	***Coef***	***SE***	***t or z***	***Coef***	***SE***	***t or z***
FFD	Interference [ant. match]	0.00	0.02	0.26	0.00	0.02	−0.23	−0.01	0.02	−0.93
	Interference [ant. mismatch]	0.02	0.02	1.09	**0.04**	**0.02**	2.30^*^	0.02	0.02	1.55
FPRT	Interference [ant. match]	−0.01	0.02	−0.71	−0.01	0.02	−0.40	0.02	0.02	1.05
	Interference [ant. mismatch]	0.03	0.02	1.68	**0.07**	**0.02**	3.16^*^	**0.06**	**0.02**	2.79^*^
RBRT	Interference [ant. match]	−0.03	0.02	−1.46	−0.01	0.02	−0.52	0.02	0.02	1.13
	Interference [ant. mismatch]	0.02	0.02	1.24	**0.05**	**0.02**	2.07^*^	0.04	0.02	1.89
RPD	Interference [ant. match]	−0.04	0.03	−1.59	0.03	0.03	1.11	0.02	0.03	0.61
	Interference [ant. mismatch]	0.00	0.02	0.03	**0.06**	**0.03**	2.07^*^	0.03	0.03	1.04
FPRP	Interference [ant. match]	−0.11	0.13	−0.84	0.17	0.10	1.64	0.03	0.09	0.33
	Interference [ant. mismatch]	−0.17	0.13	−1.36	0.14	0.10	1.40	0.00	0.09	0.00
TFT	Interference [ant. match]	0.03	0.02	1.40	0.01	0.02	0.26	0.04	0.02	1.72
	Interference [ant. mismatch]	**0.05**	**0.02**	2.52^*^	0.04	0.02	1.80	0.02	0.02	1.04
RRT	Interference [ant. match]	0.06	0.03	1.84	0.01	0.04	0.33	0.02	0.04	0.41
	Interference [ant. mismatch]	0.03	0.03	0.77	0.04	0.04	0.90	−0.04	0.04	−1.06

Statistically significant (α= *0.05*) effects are marked with an asterisk and highlighted in bold.

The main effect of antecedent (longer reading times or a higher proportion of regressions in antecedent-mismatch conditions) was significant across regression-related measures (RPD, RBRT, FPRP) and late measures (TFT, RRT). In RPD and RBRT, the effect of antecedent started already at the pre-critical region and remained significant at the reflexive and the post-critical region. In FPRP, the effect was significant at the reflexive only. In TFT, the effect also started at the pre-critical region and continued to be significant at the reflexive. In RRT, the effect reached significance only at the pre-critical region.

The main effect of interference (longer reading times or higher proportion of regressions in distractor-match conditions) reached significance across first-pass, regression-related and late measures. In RPD and FPRP, the effect reached significance at the reflexive itself, in FPRT and RBRT at the post-critical region and in TFT at the pre-critical region.

The interaction between antecedent and interference reached significance at the reflexive across first-pass and regression-related measures (FFD, FPRT, RBRT). In RBRT, this interaction was already present at the pre-critical region. Pairwise comparisons revealed that the interference effect was driven by the antecedent-mismatch conditions: Within antecedent-mismatch conditions, an inhibitory interference effect was observed across first-pass, regression-related and late measures (FFD, FPRT, RBRT, RPD, TFT).^6^ In FFD, FPRT, RBRT, and RPD, the effect reached significance at the reflexive itself and, in FPRT, continued to be significant at the post-critical region. In TFT, the effect reached significance at the pre-critical region only. Within antecedent-match conditions, the interference effect did not reach significance in any measure or region.

Moreover, the models revealed that the higher frequency of the antecedent led to a significant slowdown at the reflexive in regression-based measures (RPD: estimate = 0.03, *SE* = 0.01, *t* = 2.12; RBRT: estimate = 0.02, *SE* = 0.01, *t* = 2.00) and RRT (estimate = 0.05, *SE* = 0.02, *t* = 2.76). Frequency of the distractor, in contrast, did not affect reading times at the reflexive in any measure.

One potential issue with the data analysis reported here is the so-called multiple-testing problem, that is, testing more than one dependent variable but keeping the significance threshold α unchanged at 0.05. Although in the field of psycholinguistics it is uncommon to apply an α-level correction when multiple eye-tracking measures are analyzed, we applied a Bonferroni correction to the α-level (Bonferroni, 1936; Dunn, 1959, 1961) and checked whether the effects reported above remained significant under this more conservative analysis. This is important in order to reduce the Type I error probability because, as has been noted for example by Ioannidis (2005), false positives are a serious issue in empirical science and in psychological science in particular (Simmons et al., 2011). With respect to reading studies, von der Malsburg and Angele, “The elephant in the room: False positive rates in standard analyses of eye movements in reading” (unpublished manuscript) recently showed by means of Monte Carlo simulations that testing multiple eye-tracking measures leads to a more dramatic increase of Type I errors as compared to what had been generally believed in the field. Von der Malsburg and Angele therefore recommend to apply a Bonferroni correction to the α-level. Given that we have analyzed seven dependent variables, the Bonferroni correction yields a corrected α-level of 0.007, which corresponds to an approximate *t*-value of ± 2.69.^7^ With this adjusted α-level, the main effect of antecedent remained significant in RBRT at the pre-critical region and in RPD at the reflexive and at the post-critical region. The main effect of interference reached significance in FPRT at the post-critical region and in TFT at the pre-critical region. The interaction between antecedent and interference was significant in FPRT at the reflexive. In pairwise comparisons, the interference effect in antecedent-mismatch conditions in FPRT at the reflexive and at the post-critical region remained significant. The antecedent-frequency effect reached the Bonferroni-corrected significance threshold in RRT, but not in RPD and RBRT. In sum, although the Bonferroni correction and the considerable loss in statistical power that goes along with it makes some effects lose statistical significance, the overall pattern of results remains unchanged: An early interference effect at the reflexive present only within antecedent-mismatch conditions, an effect of antecedent in regression-related dependent variables starting already at the verb preceding the reflexive and an effect of antecedent-frequency at the reflexive.

### 2.3. Discussion

Comprehension questions required participants to correctly identify the reflexive's antecedent and to select it from four response options. Although participants could choose the option “not sure,” they were highly likely to choose the antecedent even if it was inanimate and hence a semantically illicit antecedent. This shows that in their final interpretation of the reflexive they gave structural information a higher priority than semantic information. In antecedent-match conditions only, the distractor was chosen more often in case it was animate. But, crucially, this higher proportion of distractor choices was at the cost of choices of the unrelated noun, not of the antecedent. From this pattern we conclude that the observed effect reflects *offline* interference, i.e., an effect driven by meta-linguistic considerations at the moment of answering the comprehension question. If, in contrast, the effect reflected retrieval interference during the actual sentence reading, i.e., online effects, it would be expected to manifest itself in a higher proportion of misretrievals of the distractor leading to a lower proportion of choosing the *antecedent*, not the unrelated noun, because the latter is only introduced in the question.

The analyses of eye movements firstly showed that the presence of an animate distractor led to a processing slowdown (i.e., inhibitory interference) in antecedent-mismatch conditions. This slowdown was observed across first-pass, regression-related and late measures. In the more conservative analysis with Bonferroni-corrected significance threshold, this slowdown remained reliable in FPRT. In antecedent-match conditions, this interference effect did not reach significance. This pattern cannot be explained by either of the two accounts under discussion: The parser's sensitivity to the presence of an animate distractor cannot be accounted for by a structure-based retrieval mechanism. ACT-R cannot explain the results either since, in its current implementation, ACT-R predicts facilitatory rather than inhibitory interference in antecedent-mismatch conditions caused by a higher proportion of misretrievals of an animate distractor. Kush and Phillips (2014) also found inhibitory interference in antecedent-mismatch conditions in a self-paced reading experiment on Hindi reciprocals. They explain this effect in terms of interference that occurs during a later repair process of the ungrammatical sentence rather than at the moment of retrieval. Crucially, in Kush and Phillips (2014)'s experiment, the interference effect reached marginal significance only two words after the reciprocal. For the present experiment, their explanation seems implausible since the interfere effect reaches significance already in first-pass measures at the reflexive.

Second, we did *not* find any interference effects in the antecedent-match conditions. Although these results are statistically inconclusive, it is worth mentioning that this is consistent with the findings of Chen et al. (2012), who found interference effects in non-locally bound *ziji* but failed to find effects in locally-bound *ziji*.

Third, we observed a slowdown due to an inanimate antecedent in regression-related and late measures. This grammaticality effect is in line with both structure-based retrieval and the ACT-R model. In contrast to the interference effect, this effect is most pronounced at the pre-critical region. We will discuss possible explanations for this early appearance of the effect in the Discussion of Experiment 2.

Fourth, we found that lower frequency of the antecedent led to faster reading times at the reflexive. This effect might be explained by a low-frequency encoding advantage. It has been shown that the lower frequency of a word leads to a better memory encoding which results in a faster retrieval at a later point in time (Diana and Reder, 2006). Thus, low frequency antecedents might be better encoded in memory leading to a facilitated retrieval when reaching the reflexive, which shows the more prominent role of the antecedent in the retrieval process. Indeed, this facilitation due to infrequent antecedents replicates findings from English pronouns. In an eye-tracking-while-reading experiment, Van Gompel and Majid (2004) found faster FFD and FPRT at the region following the reflexive as a function of lower frequency of the antecedent.

One potential concern with the present results might be that task-related influences on interference cannot be ruled out. One of the two comprehension questions following the experimental sentences targeted the reflexive-antecedent dependency, which—in particular in the ungrammatical conditions—might have caused readers to spend some additional reading time to rule out the animate distractor. This would explain the observed inhibitory interference in the target-mismatch conditions. In the design of the experiment, we had addressed this potential issue by including ungrammatical fillers containing *ziji* with questions that did not target the reflexive-antecedent dependency. Moreover, participants had the option to answer “not sure,” which allowed them not to assign any meaning to an ungrammatical sentence. If task-specifics had been an influential factor, they would most probably be reflected in repair attempts that are triggered by unexpectedly retrieving an inanimate antecedent. However, the interference effect reached significance already in FFD and FPRT. Based on a large-scale review of eye movements in reading, Clifton et al. (2007) have suggested that early measures like FFD or FPRT are unlikely to reflect repair processes since across studies, repair or reanalysis effects are typically observed in regression-related or later-pass reading measures. To the extent that Clifton et al. (2007)'s claim is correct, we can conclude that repair processes caused by the task-demands are unlikely to explain the observed results.

## 3. Experiment 2

This experiment extended Experiment 1 in several aspects. First, it examined proactive rather than retroactive interference; second it examined the influence of distractor items that are not a syntactic part of the sentence itself but presented as memory load; third, we tested the influence of syntactic locality on the retrieval and its interaction with interference. Previous studies report a processing slowdown in case *ziji* is non-locally bound compared to locally bound *ziji* (Gao et al., 2005; Li and Zhou, 2010; Dillon, 2011; Chen et al., 2012; Dillon et al., 2014). In the present experiment, we aimed at replicating this locality effect and investigating whether interference effects are modulated by locality of the reflexive binding.

In a dual-task paradigm, similar to Van Dyke and McElree (2006), participants were asked to remember three animate or three inanimate distractor nouns while reading a sentence containing an either locally or non-locally bound reflexive. This resulted in a 2 × 2 design, with locality (local vs. non-local) and the distractors' animacy (animate vs. inanimate) as factors. Conditions with animate distractors are labeled as *distractors-match* and conditions with inanimate distractors as *distractors-mismatch*.

The structure-based account predicts no effect of animacy of the distractor nouns held in memory. In contrast, the standard ACT-R cue-based retrieval model predicts an inhibitory interference effect due to animacy of the distractors: retrieval times at the reflexive are predicted to be longer in distractors-match conditions. Moreover, ACT-R predicts a main effect of locality with non-local conditions being read slower. This prediction does not follow from the cue-based nature of the retrieval mechanism but rather from the ACT-R assumption of decay: The more recent, i.e., the local, antecedent has a higher level of activation than the non-local antecedent when reaching the reflexive. This difference in activation is predicted to be reflected in both, retrieval times and comprehension accuracies. Since this predicted locality effect is unrelated to the set of cues used for retrieval, the structure-based cue-based retrieval account (i.e., the ACT-R model with only structural features used as retrieval cues) makes the same prediction. Moreover, a structure-based serial search mechanism that first checks the local subject position and subsequently the non-local subject as proposed by Dillon (2011) and Dillon et al. (2014) for the processing of Mandarin *ziji* also predicts a processing slowdown in non-local conditions.

**Table:** 

(3)	a. **Local binding**									
	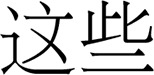	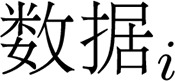	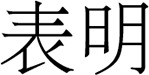	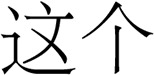	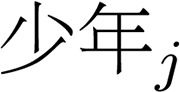	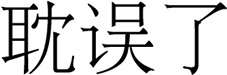	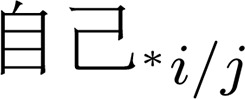	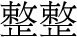	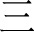	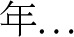
	Zhe-xie	**shuju**_*i*_	biaoming	[zhe-ge	**shaonian**_*j*_	danwu-le	ziji_*i/*j*_	zhengzheng	san	nian]…
	*this*-cl	*data*	*demonstrate*	*this*-cl	*youngster*	*hinder*-asp	*self*	wholly	three	years…
	*These data demonstrate that this youngster hindered himself three whole years…*									
	b. **Non-local binding**									
	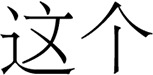	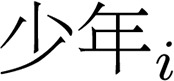	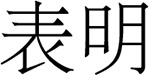	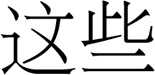	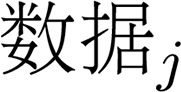	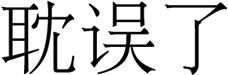	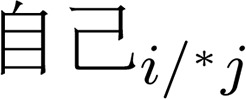	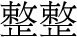	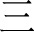	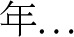
	Zhe-ge	**shaonian**_*i*_	biaoming	[zhe-xie	**shuju**_*j*_	danwu-le	**ziji**_*i*/*j_	zhengzheng	san	nian]…
	*this*-cl	*youngster*	*demonstrate*	*this*-cl	*data*	*hinder*-asp	*self*	*wholly*	*three*	*years*…
	*This youngster demonstrates that these data hindered him three whole years…*									

### 3.1. Materials and method

#### 3.1.1. Materials

We tested 36 experimental sentences^8^ which consisted of a super-ordinate clause and an embedded clause containing the reflexive *ziji* as direct object. The locality factor of the antecedent-reflexive dependency was achieved by manipulating animacy of the local subject (i.e., the subject of the embedded clause) and the non-local subject (i.e., the subject of the superordinate clause): in the local conditions, the local subject was animate and the non-local subject was inanimate (see 3a) while in the non-local conditions, the local subject was inanimate and the non-local subject was animate (see 3b). Since *ziji* requires its antecedent to be animate, this design ensured that in the local conditions, *ziji* was bound by the local subject whereas in the non-local conditions it was bound by the subject of the superordinate clause. Similar to Experiment 1, the reflexive was followed by a spillover region consisting of four characters that formed a frequency phrase or a durational phrase. Each sentence was followed by a yes/no-comprehension question that probed for the correct binding of the reflexive. Seventy-two filler sentences containing reflexives and pronouns in varying syntactic positions were presented with memory load words of varying part-of-speech.

*Pretest*. In order to verify that speakers of Mandarin indeed bind the reflexive to the local subject/the superordinate subject in the local/non-local condition, respectively, we presented 40 native speakers of Mandarin recruited at Beijing Normal University with the experimental sentences in form of a paper-based questionnaire against payment of 25 RMB (approximately 3 EUR). Ninety filler sentences containing *ziji* in various syntactic positions were included. Participants were instructed to circle the word in the sentence *ziji* referred to, or, in case they found that no antecedent was available in the sentence, to write down which entity *ziji* referred to.

*Results*. Overall, 90.4% of all trials were answered as we had expected: In the local conditions, the animate local subject was chosen as antecedent and in the non-local conditions the animate matrix subject was selected. In the local conditions, accuracy was lower (85.1%) than in the non-local conditions (95.6%). A syntactic classification of the incorrect answers is provided in the Appendix.

#### 3.1.2. Participants and procedure

This experiment was conducted in the same laboratory as Experiment 1. One hundred thirty native speakers of Mandarin with normal or corrected-to-normal vision participated in the experiment against payment of 60 RMB (approximately 7 EUR). The general experimental set-up was the same as in Experiment 1. The experiment was split into two experimental sessions (40–70 minutes per session) conducted on two subsequent days. At the beginning of each trial, the three distractors were shown on the screen one below another for 3 seconds. When the words disappeared, the test sentence was displayed. After having finished reading the sentence, the comprehension question was presented. After having answered the comprehension question, participants were asked to serially recall the distractors: The three distractors together with three unrelated items (similarly animate or inanimate nouns) were displayed simultaneously on the screen as a numbered list in randomized order. Participants were asked to choose the distractors in their correct order from this list.

### 3.2. Results

For all dependent variables, we fit two sets of contrasts; the first tested for main effects of locality (local conditions coded as −0.5; non-local conditions coded as +0.5) and interference (animate distractors coded as +0.5; inanimate distractors coded as −0.5) and their interaction; in the second model pairwise comparisons of memory load nested within each level of locality were applied. In addition, experimental session (first vs. second session) was coded with sum-contrasts and its interaction with the other effects were included as predictors. All models were fit with random intercepts for items and participants, no random slopes were fit since they led to convergence failure in most of the models.

#### 3.2.1. Comprehension questions

Mean accuracy scores by experimental condition are shown in Table 6. None of the comparisons reached statistical significance.^9^

**Table 6:** **Experiment 2: Comprehension question response accuracy in percentage by experimental condition**.

***Locality***	***Distractors***	**Accuracy**
*local*	*match*	67.1
	*mismatch*	68.7
*non-local*	*match*	71.8
	*mismatch*	71.9

#### 3.2.2. Memory recall

Mean serial and non-serial recall accuracies for each of the three distractors and total serial and non-serial recall accuracy (i.e., all distractors recalled correctly) are presented in Table 7. In the statistical analyses of total serial recall accuracy none of the comparisons reached significance. In the analyses of total non-serial accuracies, the interaction between animacy of the distractors and locality was significant (estimate = −0.22, *SE* = 0.10, *z* = −2.21, *p* < 0.05). Pairwise comparisons revealed that this interaction was driven by a significant effect of distractors (lower recall accuracy of animate distractors) that was present only in local conditions (estimate = −0.30, *SE* = 0.14, *z* = −2.25, *p* < 0.05).

**Table 7:** **Experiment 2: Mean serial and non-serial recall accuracy in percentage of the three memory load words separately and total accuracy in percentage presented by experimental condition**.

	**Serial accuracy**				**Non-serial Accuracy**			
***Locality***	***Local***		***Non-local***		***Local***		***Non-local***	
***Distractors***	***Match***	***Mism***.	***Match***	***Mism***.	***Match***	***Mism***.	***Match***	***Mism***.
1st word correct	85	83	85	85	92	94	94	94
2nd word correct	79	75	81	79	93	94	94	93
3rd word correct	82	78	83	82	90	91	92	91
Total correct	68	67	71	69	77	81	82	80

#### 3.2.3. Eye movements

The same log-transformed dependent variables as in Experiment 1 were analyzed at the reflexive, the verb preceding it (pre-critical), and the spillover material (post-critical). As in the analysis of Experiment 1, trials were excluded when the continuous variable on which the analysis was carried out was zero. First-pass fixations occurred at the pre-critical region, the reflexive, and the spillover region in 86, 50, and 85% of the trials, respectively. Re-readings were recorded in 55, 25, and 36% of the trials at pre-critical region, the reflexive, and the spillover region, respectively. Mean reading times with standard errors for each dependent variable are provided in Table 8.

**Table 8:** **Experiment 2: Means and standard errors of raw first-fixation duration, first-pass reading time, right-bounded reading time, regression-path duration, total fixation time, re-reading time in ms, and first-pass regression probability in percentages at the pre-critical region, the reflexive and the post-critical region**.

	**Pre-critical**				**Reflexive**				**Post-critical**			
***Locality***	***Local***		***Non-local***		***Local***		***Non-local***		***Local***		***Non-local***	
***Distractors***	***Match***	***Mism***.	***Match***	***Mism***.	***Match***	***Mism***.	***Match***	***Mism***.	***Match***	***Mism***.	***Match***	***Mism***.
FFD	267 (5)	268 (5)	267 (5)	270 (5)	251 (6)	239 (5)	240 (5)	244 (6)	257 (6)	255 (5)	253 (5)	258 (6)
FPRT	342 (9)	341 (9)	351 (9)	343 (9)	260 (7)	245 (6)	250 (6)	254 (7)	325 (10)	320 (10)	322 (9)	320 (9)
RBRT	398 (11)	409 (11)	433 (12)	447 (13)	278 (7)	259 (6)	263 (7)	277 (8)	378 (11)	375 (12)	383 (12)	375 (11)
RPD	575 (25)	573 (26)	596 (24)	638 (28)	486 (28)	419 (27)	448 (29)	484 (32)	636 (35)	628 (36)	667 (41)	710 (47)
FPRP	23 (2)	20 (2)	25 (2)	28 (2)	18 (1)	14 (1)	16 (1)	16 (1)	24 (2)	24 (2)	25 (2)	25 (2)
TFT	683 (25)	666 (22)	737 (26)	763 (28)	396 (14)	354 (13)	377 (14)	379 (14)	501 (19)	491 (18)	508 (19)	479 (17)
RRT	626 (33)	592 (28)	645 (33)	704 (34)	352 (22)	365 (26)	360 (24)	345 (22)	432 (29)	414 (30)	468 (32)	413 (26)

In the calculation of standard errors of continuous dependent variables, between-participants variance has been removed using the Cousineau (2005) normalization with Morey (2008)'s correction. For continuous variables, trials with a 0 as value of the respective variable have been excluded.

The output of the linear-mixed models is summarized in Tables 9 and 10. The effect of experimental session was significant across regions and measures: Participants read faster in their second experimental session.^10^ The main effect of locality reached significance across regression-based and later-pass measures (RBRT, RPD, FPRP, RRT, TFT) at the pre-critical region only. The main effect of interference was significant only in RRT at the post-critical region (longer RRTs when distractors were animate, i.e., inhibitory interference). The interaction between locality and interference was significant across first-pass, regression-based, and later-pass measures (FFD, FPRT, RBRT, RPD, TFT) at the reflexive. The pairwise comparisons revealed that the interaction was driven by a slowdown for animate distractors at the reflexive that was present only in local conditions. This inhibitory interference reached significance across first-pass, regression-based, and later-pass measures (FPRT, RBRT, RPD, TFT). For non-local conditions, a similar slowdown was observed only in RRT at the post-critical region.

**Table 9:** **Experiment 2: Main effects of locality and interference and their interaction at the pre-critical (*****ziji*****−1), critical (*****ziji*****), and post-critical (*****ziji*****+1) regions for the dependent variables (DVs) first-fixation duration, first-pass reading time, right-bounded reading time, regression-path duration, first-pass regression probability, total fixation time, and re-reading time**.

**DV**	**Comparison**	**Pre-critical**			**Reflexive**			**Post-critical**		
		***Coef***	***SE***	***t or z***	***Coef***	***SE***	***t or z***	***Coef***	***SE***	***t or z***
FFD	Locality	0.00	0.01	−0.06	−0.01	0.01	−0.92	0.00	0.01	−0.19
	Interference	−0.01	0.01	−0.98	0.01	0.02	0.35	−0.01	0.02	−0.58
	Locality × Interference	0.00	0.02	0.00	**0.04**	**0.02**	2.20^*^	0.02	0.02	1.21
FPRT	Locality	0.01	0.02	0.52	−0.01	0.02	−0.83	0.00	0.02	0.24
	Interference	0.00	0.02	0.26	0.01	0.02	0.63	0.00	0.02	−0.23
	Locality × Interference	0.00	0.02	−0.23	**0.04**	**0.02**	2.33^*^	0.02	0.02	0.85
RBRT	Locality	**0.08**	**0.02**	5.37^*^	−0.01	0.02	−0.34	0.01	0.02	0.36
	Interference	−0.01	0.02	−0.49	0.01	0.02	0.61	0.00	0.02	0.01
	Locality × Interference	0.00	0.02	0.03	**0.06**	**0.02**	3.21^*^	0.02	0.02	0.79
RPD	Locality	**0.11**	**0.02**	5.15^*^	0.03	0.03	1.10	0.02	0.03	0.78
	Interference	0.00	0.03	−0.08	0.04	0.03	1.36	−0.01	0.03	−0.30
	Locality × Interference	0.02	0.03	0.71	**0.07**	**0.03**	2.16^*^	0.03	0.03	0.95
FPRP	Locality	**0.46**	**0.08**	5.80^*^	0.11	0.09	1.19	0.05	0.08	0.62
	Interference	0.04	0.10	0.43	0.15	0.11	1.34	0.00	0.09	0.02
	Locality × Interference	0.14	0.10	1.41	0.09	0.11	0.83	−0.01	0.09	−0.12
TFT	Locality	**0.10**	**0.02**	5.55^*^	−0.01	0.02	−0.31	0.00	0.02	−0.15
	Interference	0.01	0.02	0.57	0.05	0.02	1.92	0.02	0.02	1.01
	Locality × Interference	0.01	0.02	0.34	**0.08**	**0.02**	3.10^*^	0.00	0.02	−0.16
RRT	Locality	**0.10**	**0.03**	3.71^*^	−0.01	0.04	−0.23	0.06	0.04	1.73
	Interference	−0.02	0.03	−0.55	0.00	0.05	0.04	**0.09**	**0.04**	2.10^*^
	Locality × Interference	0.07	0.03	1.92	−0.03	0.05	−0.64	−0.04	0.04	−0.89

Statistically significant (α= *0.05*) effects are marked with an asterisk and highlighted in bold.

**Table 10:** **Experiment 2: Interference effect nested within each level of locality (local vs. non-local) at the pre-critical (*****ziji*****−1), critical (*****ziji*****), and post-critical (*****ziji*****+1) regions for the dependent variables (DVs) first-fixation duration, first-pass reading time, right-bounded reading time, regression-path duration, first-pass regression probability, total fixation time, and re-reading time**.

**DV**	**Comparison**	**Pre-critical**			**Reflexive**			**Post-critical**		
		***Coef***	***SE***	***t or z***	***Coef***	***SE***	***t or z***	***Coef***	***SE***	***t or z***
FFD	Interference [local]	−0.01	0.02	−0.69	0.04	0.03	1.79	0.01	0.02	0.45
	Interference [non−local]	−0.01	0.02	−0.69	−0.03	0.02	−1.31	−0.03	0.02	−1.26
FPRT	Interference [local]	0.00	0.03	0.02	**0.06**	**0.03**	2.09^*^	0.01	0.03	0.44
	Interference [non−local]	0.01	0.03	0.35	−0.03	0.03	−1.21	−0.02	0.03	−0.76
RBRT	Interference [local]	−0.01	0.03	−0.33	**0.07**	**0.03**	2.70^*^	0.02	0.03	0.57
	Interference [non−local]	−0.01	0.03	−0.37	−0.05	0.03	−1.83	−0.02	0.03	−0.55
RPD	Interference [local]	0.02	0.04	0.44	**0.11**	**0.05**	2.49^*^	0.02	0.04	0.46
	Interference [non−local]	−0.02	0.04	−0.56	−0.03	0.05	−0.57	−0.04	0.04	−0.89
FPRP	Interference [local]	0.18	0.14	1.23	0.24	0.15	1.52	−0.01	0.13	−0.07
	Interference [non−local]	−0.09	0.13	−0.74	0.06	0.15	0.36	0.01	0.13	0.10
TFT	Interference [local]	0.02	0.03	0.64	**0.12**	**0.03**	3.56^*^	0.02	0.03	0.60
	Interference [non−local]	0.00	0.03	0.16	−0.03	0.03	−0.83	0.03	0.03	0.82
RRT	Interference [local]	0.05	0.05	0.94	−0.03	0.07	−0.42	0.05	0.06	0.87
	Interference [non−local]	−0.08	0.05	−1.79	0.03	0.06	0.49	**0.13**	**0.06**	2.09^*^

Statistically significant (α=*0.05*) effects are marked with an asterisk and highlighted in bold.

As we did for Experiment 1, we checked which of the observed effects remained significant with a Bonferroni-corrected significance threshold. Given seven dependent variables, the corrected α-level is 0.007, which corresponds to an approximate *t*-value of ± 2.69.^11^ The significance of the main effect of locality was not affected by this correction in any dependent variable, it remained significant at the pre-critical region in RBRT, RPD, FPRP, TFT, and RRT. The main effect of interference at the post-critical region in RRT did not reach the adjusted significance threshold. The interaction between locality and interference remained significant at the reflexive in RBRT and TFT, but did not reach significance anymore in FFD, FPRT, and RPD. In pairwise comparisons, the interference effect in local conditions at the reflexive remained significant in RBRT and TFT, but did not reach the significance threshold anymore in FPRT and RPD. The interference effect in non-local conditions that was observed at the post-critical region did not reach the adjusted significance threshold. In sum, the main effect of locality as well as the interference effect in locally bound *ziji* remained significant in various dependent variables even with an adjusted α-level. The interference effect in non-local conditions, in contrast, was not reliable under the corrected α-level.

### 3.3. Discussion

In the comprehension questions, no evidence for an interference effect was found. In the memory recall task, in contrast, we found that, in local conditions only, animate words were more difficult to recall than inanimate words.

First, we found evidence for a processing slowdown associated with the non-local binding of the reflexive. This locality effect replicates findings from SAT (Dillon, 2011; Dillon et al., 2014), ERP (Li and Zhou, 2010; Dillon, 2011), cross-modal priming (Liu, 2009), and self-paced reading (Chen et al., 2012), and is accounted for by the ACT-R model, no matter whether the set of retrieval cues is unconstrained or limited to structural cues. The structure-based serial search as proposed by Dillon (2011) and Dillon et al. (2014) is also in line with the observed locality effect. However, it is not fully clear why this locality effect appears at the verb preceding the reflexive rather than at the reflexive itself. One explanation would be a preview effect. Alternatively, it might be the case that the observed effect does not reflect locality of the reflexive binding but rather the verb's preference for an animate subject since the locality manipulation is achieved by having the local subject either animate or inanimate. Along the same lines, one could explain why in Experiment 1, the effect of animacy of the antecedent becomes significant already at the verb preceding the reflexive. A strong indication that the observed effect at the verb indeed reflects the verb's preference for an animate subject comes from a re-analysis of the self-paced reading data reported by Chen et al. (2012), where the locality manipulation was also achieved by varying the animacy of the local and non-local subjects, and the main clause verb also directly preceded the reflexive *ziji*. Chen et al. (2012) analyzed only the region containing the reflexive and the regions *following* the reflexive, but not the verb *preceding* the reflexive. Re-analyzing their data at the verb region revealed that the locality effect in their data was already significant at the verb (*t* = 2.5). As preview effects are ruled out as an explanation in self-paced reading, and given the high structural similarity of our experimental materials to the ones used by Chen et al. (2012), we conclude that the effect observed at the verb in Experiment 2 is most likely due to an animacy preference of the verb. Given this—admittedly unforeseen—confounding animacy preference of the verb, we cannot draw any conclusions about the actual locality manipulation. A potential locality effect might have been masked by the stronger effect of animacy preference: when reaching the verb in the non-local conditions, readers are highly likely to re-read the previous material to overcome the difficulty associated with the verb's inanimate subject, as indicated by the highly significant effects in FPRP, RPD, and RBRT. This leads to activation of the preceding materials in the non-local conditions *directly before* reaching the reflexive, which, in turn, might have canceled out a locality effect at the reflexive. Therefore, we conclude that our data is inconclusive with respect to the locality manipulation.

Second, we found clear evidence for inhibitory interference, but the time-course of this effect was different for local and non-local conditions. In local conditions, animate distractors led to a slowdown across first-pass, regression-based, and late eye-tracking measures at the reflexive itself. Even with a Bonferroni corrected significance threshold of α = 0.007, this effect remained significant in RBRT and TFT. In FPRT and RPD, the inhibitory interference effect did not survive Bonferroni correction. However, since these measures numerically pattern with other measures—especially with RBRT, which is closely related—it could reflect a real effect. In non-local conditions, the interference effect appeared only later in processing (in RRT at the post-critical region). However, with Bonferroni adjusted significance threshold, this effect was not reliable. In sum, the observed interference pattern extends the findings of Experiment 1 in two respects. First, Experiment 2 shows that locally bound *ziji* is subject to early interference even in case a fully cue-matching antecedent is available. The difference to Experiment 1, where the interference effect did not reach significance in antecedent-match conditions, might be explained by the different experimental paradigms: rehearsal of the distractors during reading might cause stronger interference than the sentence-internal manipulation of Experiment 1. Second, the interference profile in non-locally bound *ziji* differs from the one in locally bound *ziji* in the sense that in non-local conditions no early effect was found, but there is weak evidence for a late effect. Although the late effect in non-local conditions was not significant under Bonferroni correction, there is reason to believe in this effect when viewed against the background of previous findings by Chen et al. (2012), who found an inhibitory interference effect in non-local *ziji*.

The observed interference effects are not compatible with a structure-based retrieval mechanism since no effect of the distractors is predicted. The ACT-R model, in contrast, can account for the inhibitory interference effect. However, ACT-R is unable to explain the delayed appearance of the effect in non-local conditions.

A possible explanation for the different interference patterns in local vs. non-local conditions could be that qualitatively different mechanisms are involved in the processing of locally and non-locally bound *ziji*. In the syntactic literature, it has been proposed that only the locally bound *ziji* should be regarded as a reflexive pronoun whereas non-locally bound *ziji* should be regarded as a logophoric pronoun which is subject to pragmatic and discourse constraints rather than to purely syntactic binding principles (Huang and Liu, 2001; Huang, 2002). One prominent argument favoring this idea of two lexically different instances of *ziji* are blocking effects observed in long-distance *ziji* but not in local *ziji* (Huang, 1984, 2002; Tang, 1989; Huang and Tang, 1991; Xue et al., 1994; Pan, 2000). A qualitative distinction between locally bound *ziji* and non-local *ziji* has also been proposed in the psycholinguistic literature. Based on previous work by Gao et al. (2005), Liu (2009) conducted a cross-modal priming experiment using sentences in which both a local and a non-local animate antecedent were present (i.e., globally ambiguous sentences in terms of binding) and manipulated stimulus-onset asynchrony (0 ms, 160 ms, 370 ms). When the probe was presented directly after the offset of the reflexive (SOA = 0 ms), a semantic priming effect for probes related to the local antecedent but not for probes related to the non-local antecedent was observed. At an SOA of 160 ms, in contrast, the pattern was reversed: There was a priming effect for probes that were semantically related to the non-local antecedent, but no priming effect for probes related to the local antecedent. At an SOA of 370 ms, both the local and non-local antecedent elicited a semantic priming effect. Liu (2009) interpreted these results as evidence for *ziji* being bound by the local subject in a first stage of processing and by the non-local subject in a second stage of processing, whereas in the final stage, both bindings are possible. Along the same lines, Dillon (2011) and Dillon et al. (2014) suggested that the parser tries to first access the local subject and only at a later stage accesses non-local antecedent positions. Such a temporal delay for the triggering of the retrieval of a non-local antecedent would indeed predict the pattern observed in Experiment 2: In the local conditions, the retrieval is triggered immediately at the moment when the reflexive is first encountered. The interference effects associated with this retrieval therefore appear already in early measures at the reflexive. In non-local conditions, in contrast, the retrieval of the non-local antecedent is triggered only after a certain delay, which causes the interference effects to occur only in RRT at the spillover region.

## 4. An extended cue-based retrieval model

As has been pointed out in the experimental discussions, the interference effects observed in the experiments presented here are not compatible with structure-based accounts. The current implementation of the standard cue-based retrieval model in ACT-R (Lewis and Vasishth, 2005) cannot explain the observed patterns either. In particular, standard cue-based retrieval is unable to explain (i) why there is an effect in antecedent-match conditions in Experiment 2 but not in Experiment 1, and (ii) why there is inhibitory interference observed in antecedent-match conditions in Experiment 1. We propose an explanation of the observed patterns by adding two independently motivated assumptions to standard cue-based retrieval: that (i) similarity-based interference is modulated by *distractor prominence* and that (ii) *cue confusion* can lead to similarity-based interference between non-similar items. As discussed earlier, the difference in the interference profiles of local and non-local *ziji* might be due to a qualitative difference in processing mechanisms and was therefore not included in our modeling.

### 4.1. Principle 1: prominence

In Experiment 1, we found an interference effect in antecedent-mismatch conditions but not in antecedent-match conditions. According to Wagers et al. (2009), this is an expected prediction of cue-based retrieval and, in the context of subject-verb number attraction phenomena, the authors named it “grammatical asymmetry.” Their intuitively plausible explanation was that a perfectly matching antecedent (as is the case in antecedent-match conditions) must clearly outcompete a partially matching distractor, while more interference is caused when both antecedent and distractor are only partially matching candidates.

Simulations with the current ACT-R implementation (Lewis and Vasishth, 2005) revealed that the latter does not predict such asymmetry (for details, see Engelmann et al., 2015, and our forthcoming paper Engelmann, Jäger, and Vasishth, “Confusability of retrieval cues in dependency resolution: A computational model,” manuscript in preparation)—at least not in a principled way: It is possible to adjust ACT-R's parameters to permanently reduce similarity-based interference. However, this would leave unexplained why in some cases effects in antecedent-match conditions do appear (see the General Discussion for details). Standardly, ACT-R predicts interference effects in match and mismatch conditions. We therefore extended the ACT-R model with a *prominence principle* that scales similarity-based interference in relation to the difference in activation between antecedent and distractor.

In standard ACT-R, a memory item *i* receives an amount of spreading activation *S*_*ji*_ for each retrieval cue *j* it matches. This activation is reduced relative to the number of distractors that match the same retrieval cue *j* (this number is called the *fan*_*ji*_):

(1) $$S_{ji}=S-\text{ln}(fan_{ji})$$

where *S* is the *maximum associative strength* parameter (*MAS*), which defaults to 1.

In our model, the *fan*_*ji*_ is transformed into *fan*′_*ji*_ by a *prominence correction*, that takes into account the distractors' relative activation:

(2) $$fan^{\prime }{}_{ji}=\{\begin{array}{ll} \frac{1}{1+e^{-C(x_{0}-Diff)}}\times fan_{ji}, & \text{if }C>0\\ fan_{ji}, & \text{otherwise} \end{array}$$

where *Diff* is the difference *A*_*i*_ − *Ā*_*Competitors*_ between the target activation *A*_*i*_ and the mean activation of all competitor items associated with cue *j*. The *prominence correction factor C* scales the steepness of the logistic *prominence correction* function and should not vary within the same model. In our simulations, we set it to 5. The function's *offset x*_0_ is fixed at 1.3, which means that *fan*′_*ji*_ is 0.5 × *fan*_*ji*_ at an activation difference between target and distractor of 1.3.

**Figure 1:**
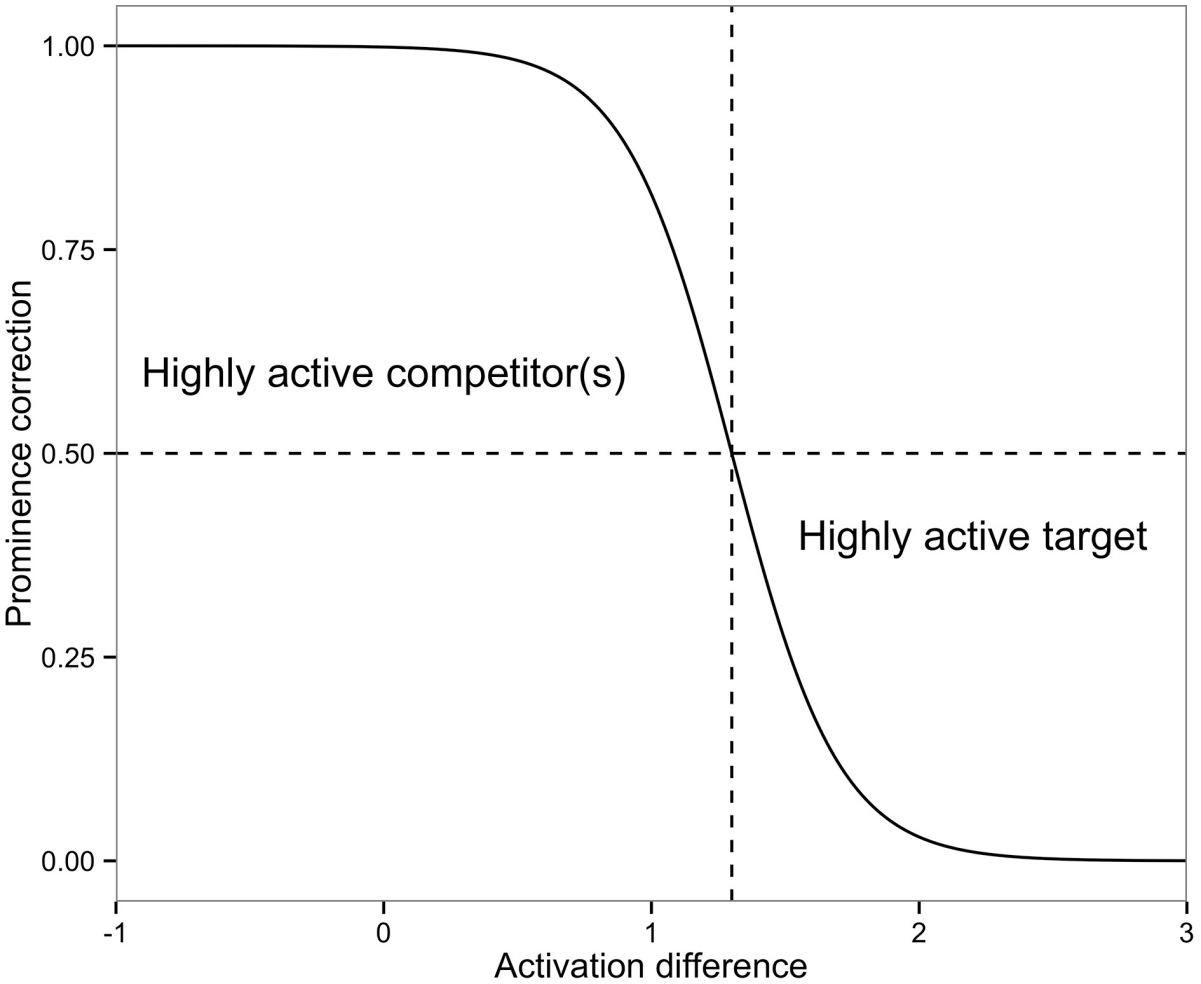
**Prominence correction by activation difference** ***Diff*** **(target − distractors) with** ***C*** **= 5 and** ***x*****_0_ = 1.3**.

Figure 1 shows the change in the multiplicative term (the *prominence correction*), that determines the relation between *fan* and its transformation *fan*′. When the target has lower activation than the mean activation of its competitors, *Diff* is negative and the prominence correction approaches 1, which implies that the fan will correspond to the standard calculation in ACT-R, and the activation of the target will be reduced by some amount. This is the case when there are highly activated distractors present: similarity-based interference occurs in this case. *Diff* will be positive when the mean activation of the competitors is relatively low. In this case, the prominence correction will be a value less than 1, and as a consequence the second term in Equation (1) will approach 0, leading to a relatively larger amount of spreading activation to the target. In other words, there will be less interference.

This implementation of a prominence principle adds two predictions to the standard cue-based retrieval model: First, there is generally less interference in antecedent-match conditions due to the presence of a highly activated fully matching antecedent. Second, similarity-based (inhibitory) interference in antecedent-match conditions is *increased* for distractors that are highly activated or when there are multiple distractors as in our Experiment 2.^12^ Distractor base-level activation could be influenced by its grammatical role (subjects are more salient or accessible than objects, Chafe, 1976; Keenan and Comrie, 1977; Brennan, 1995; Grosz et al., 1995) and by its discourse topicality (Chafe, 1976; Givón, 1983; Du Bois, 1987, 2003; Ariel, 1990; Gundel et al., 1993; Grosz et al., 1995). Other factors contributing to the salience of the distractor and hence to its base-level activation might be first mention (Gernsbacher and Hargreaves, 1988), thematic role (Arnold, 2001), contrastive focus (Cowles et al., 2007) or animacy (Fukumura and van Gompel, 2011). In effect, the prominence principle accounts for both the absence of an effect in antecedent-match conditions of Experiment 1 and the presence of an inhibitory effect in Experiment 2. Furthermore, the prominence principle predicts greater interference effects in antecedent-match conditions for distractors in more salient positions. We will relate this prediction to the literature in the General Discussion.

### 4.2. Principle 2: cue confusion

As explained in the introduction and resulting from Equation (1), similarity-based (inhibitory) interference (or the fan effect) in ACT-R only arises when multiple memory items match the same retrieval cues. Since this is not the case in the antecedent-mismatch conditions of Experiment 1, the observed inhibitory interference is incompatible with ACT-R theory. At least this seems to be the case. We argue that this assumption of incompatibility might not be justified.

In the application of cue-based retrieval to sentence comprehension, it is generally assumed that retrieval cues perfectly distinguish matching features from non-matching ones. For instance, a +*plural* cue always activates plural items and not singular items. For our first experiment, this means that +*animate* is perfectly different from +*c-com* and no similarity-based interference is predicted in antecedent-mismatch conditions where the antecedent only matches +*c-com* and the distractor only matches +*animate*. However, the language processor might not differentiate between features categorically but rather on a continuous scale of similarity. In fact, in the general ACT-R framework, features are memory items just like the items they belong to and, therefore, could be confused with each other if they have a sufficient degree of similarity. If we assume that cue-feature associations have to be learned from language experience, it follows that these associations would somehow reflect co-occurrence statistics in the language input. Consequently, cues in a retrieval specification could, depending on the retrieval-relevant context, be associated with several features to different degrees.

A co-occurrence-based account would predict differences between English reflexives and Mandarin *ziji* in the following way: *Ziji* invariably requires its antecedent to match {+*c-com*, +*animate*}, meaning that these two features frequently co-occur in the specific task of processing the Mandarin reflexive. English reflexives, on the other hand, have several alternative forms like *himself*, *herself*, *itself*, and *themselves*. All of these forms have the same structural requirement toward their antecedent but their non-structural retrieval cues vary in gender and number. The benefit of distinguishing features for number, gender, and structural relation in English reflexives results in a stronger one-to-one association between a cue and the corresponding feature. In the case of Mandarin *ziji*, however, there is no benefit from distinguishing +*c-com* and +*animate* for the task of finding the appropriate antecedent. In consequence, retrieval cues might in this case be associated with both features to some degree in a kind of *crossed association*. In relation to the retrieval specification, antecedent and distractor would appear similar in this case, although they theoretically do not share any features. This confusion-induced similarity can cause similarity-based interference as of Equation (1), predicting inhibitory effects in conditions where they would not be expected in terms of standard cue-based retrieval assumptions.

We implemented cue confusion by further adjusting the measure of similarity-based interference (the *fan*) from Equation (1) to take into account all features and their strength of association with a certain cue:

(3) $$fan_{ji}=1+\sum_{k}\left(1+Q_{jk}\right)$$

where *Q*_*jk*_ is the *associative strength* between cue value *j* and feature value *k* on a scale of [−1, 0], with −1 meaning no association and 0 representing maximum association. We assume that this association is dynamically adaptive to individual dependency environments. Equation (3) predicts that the stronger a cue-feature association the more this feature will contribute to similarity-based interference related to that cue. For example, if *Q*_*c-com*;*anim*_ for *ziji* is −0.5, the resulting fan for the +*c-com* cue would be 1.5 instead of 1 as original ACT-R would predict. This increases similarity-based interference in comparison to English reflexives, where, say, *Q*_*c-com*;*gend*_ would be standardly assumed −1, hence having a fan of 1 for each cue.

Another example of increased feature-co-occurrence are reciprocals like *each other*. In this case, the feature combination {+*c-com*, +*plural*} is invariably required. Hence, our account predicts an increased cue-confusion level in the case of English reciprocals just like in Mandarin reflexives, possibly leading to inhibitory interference in antecedent-mismatch conditions.

With the cue confusion account, we propose that task requirements (frequent co-occurrence of certain features in similar retrieval contexts) dynamically influence how cues are treated during a retrieval request. Cue confusion therefore predicts that inhibitory interference effects in antecedent-mismatch conditions should preferably be observed in constructions where cues frequently co-occur. An evaluation of these predictions beyond our own experimental results will be provided in the General Discussion.

### 4.3. Simulation results

**Figure 2:**
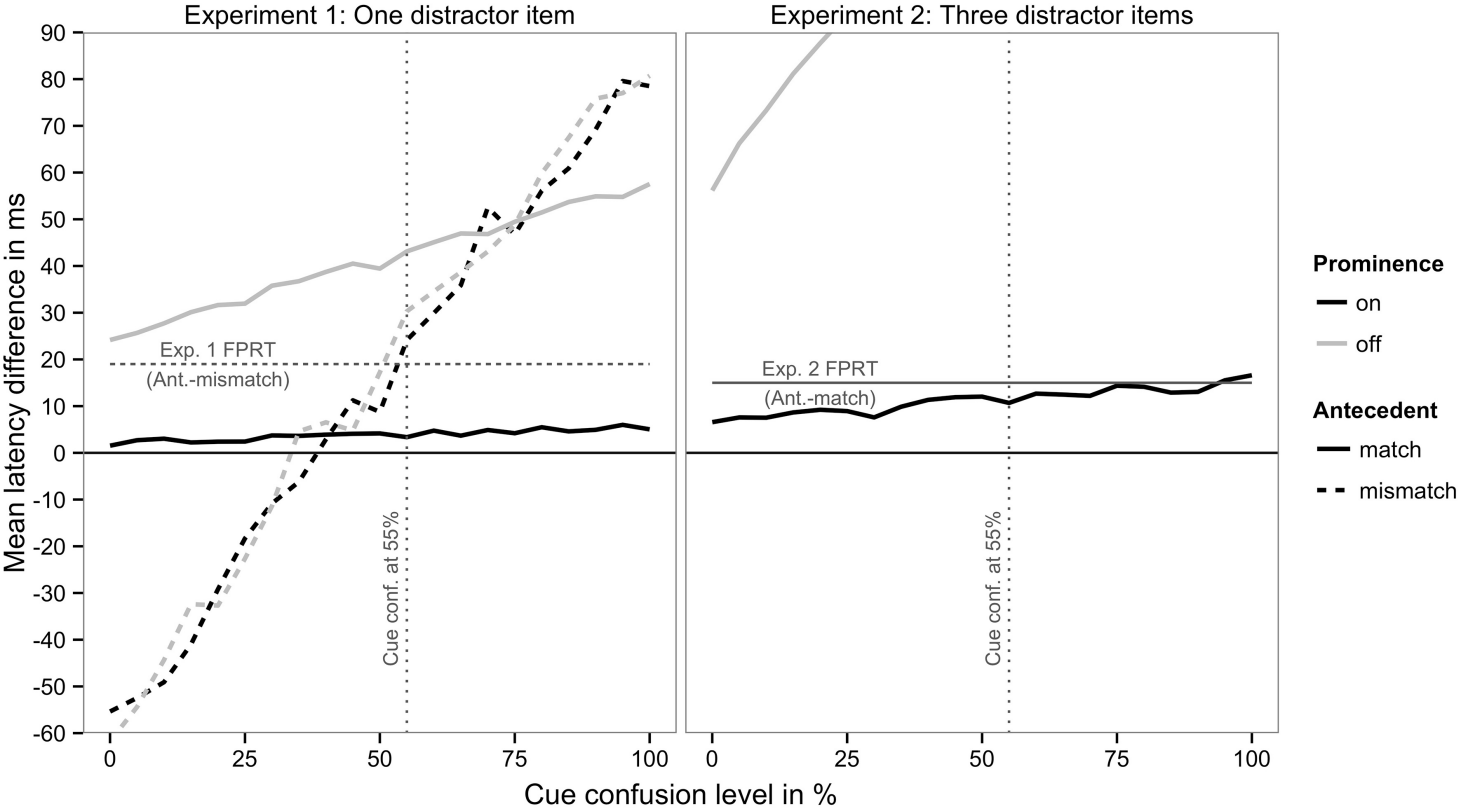
**Predicted interference effect (distractor-match − distractor-mismatch) by cue confusion level for the default model (gray lines) and the prominence model (black lines)**. The left panel shows the predicted interference for a single distractor (Exp. 1); the right panel for three distractors (Exp. 2). Solid lines represent the conditions where the antecedent matches the semantic cue, mismatch conditions are represented by dashed lines. The gray horizontal lines indicate the observed effect size in antecedent-mismatch conditions in Exp. 1 (left panel) and local antecedent-match conditions in Exp. 2 (right panel)—both in first pass reading time FPRT. The gray dotted vertical line intersects the x-axis at the estimated cue confusion value (55%) in both panels.

We report model predictions for the full range of cue confusion values. ACT-R parameters were fixed to their defaults or to values used in previous simulations (Lewis and Vasishth, 2005): latency factor *LF* = 1.5, activation noise value *ANS* = 1.5, mismatch penalty *MP* = 1.5. We compare the model predictions with empirical FPRT on *ziji* of Experiments 1 and 2. We refer to FPRT in Experiment 2 although it was not significant under Bonferroni correction. It however patterned with an effect in RBRT, which had a similar magnitude. Figure 2 plots the prediction space of a cue-based retrieval model that implements cue confusion and prominence (values represent the means of 2000 simulations each). For comparison, the predictions of a model without prominence are plotted in gray. The cue confusion level is plotted on a percentage scale, with 100% confusion meaning that both features, +*c-com* and +*animate*, are maximally associated with both the *c-com* and *animate* cues (*Q*_*c-com*;*anim*_ = 0 and *Q*_*anim*;*c-com*_ = 0). With *prominence correction factor* at 0 and *cue confusion level* at −1, the current model is equivalent to the original ACT-R model. The original model's predictions are therefore represented by the left-most points of the gray lines. The left panel shows the predictions for Experiment 1. With increasing cue confusion, the interference effect for the antecedent-mismatch conditions increases. At a confusion level of about 55% (indicated by the dotted vertical line), the model predicts an effect of the observed size in local conditions (19 ms in FPRT, indicated by the dashed horizontal line). In contrast to the original model, the prominence model predicts an interference effect close to zero for antecedent-match conditions in Experiment 1 for all cue confusion levels. This is in line with the absence of an effect in the data.

The right panel of Figure 2 shows the predictions for a similar model as the left panel, but with three distractors instead of one, simulating the conditions of Experiment 2. The inhibitory effect for antecedent-match conditions increases with cue confusion in this scenario. An effect of about the observed size (15 ms in FPRT) is predicted at the same cue-confusion level as for Experiment 1.

To summarize, the extended model with cue confusion and prominence predicts the observed data of both experiments with fixed parameters at a cue-confusion level of about 55%. More specifically, the model predicts two patterns that the original ACT-R model does not predict: (i) the absence (or near absence) of an inhibitory interference effect in the antecedent-match conditions of Experiment 1 in spite of an effect present in Experiment 2 and (ii) an *inhibitory* interference effect in antecedent-mismatch conditions in Experiment 1.

## 5. General discussion

We conducted two eye-tracking experiments in which we investigated whether the reflexive *ziji* is subject to interference effects from structurally inaccessible distractor nouns that fulfill the animacy requirement of *ziji*. In Experiment 1, where only a single distractor was present in the sentence, we found inhibitory interference in antecedent-mismatch conditions but no effect in antecedent-match conditions. In Experiment 2, where three distractors were presented as memory load, we found interference effects also in antecedent-match configurations.

These results are clear evidence against a structure-based mechanism underlying memory retrieval in human sentence parsing. The interference effects observed in Experiments 1 and 2 are incompatible with a purely structure-based retrieval mechanism. However, Sturt (2003) and Kush and Phillips (2014) have proposed a potential explanation for interference effects within the structure-based account. These authors hypothesize that, in the case of retrieval failure, a later repair process might employ a retrieval with relaxed structural restrictions, giving rise to late interference effects. This late-interference account is a plausible explanation for the effect observed in the non-local conditions of Experiment 2, where the effect occurred only in RRT at the post-critical region. However, for the effects observed in locally bound *ziji* (Experiments 1 and 2), the late-interference account appears implausible given that the effects occur already in first-pass eye-tracking measures and at the critical region.^13^ Also note that the effect reported in Kush and Phillips (2014) does not necessarily reflect late processes, since in self-paced reading experiments, it is very common that effects triggered at the critical region appear several words downstream.

The standard ACT-R model of cue-based retrieval (Lewis and Vasishth, 2005) does predict immediate interference effects but is not fully compatible with our results either. First, it predicts facilitatory rather than inhibitory interference in antecedent-mismatch conditions and, second, it cannot explain the absence of an effect in the antecedent-match conditions of Experiment 1. In fact, in the literature on reflexive processing, hardly any study can be found that reports the exact pattern predicted by the standard ACT-R model, namely inhibitory interference in antecedent-match conditions and facilitatory interference in antecedent-mismatch conditions.^14^ An approach of extending the ACT-R model in favor of a structure-based mechanism has been taken by Parker and Phillips (2014). They have proposed that structural cues are weighted higher than semantic or morphological cues, so that interference effects occur only in case of an abnormally poor match of the accessible antecedent. This is a plausible explanation for their data and offers an account for the fact that interference is hard to find in reflexives. However, with respect to our results, it neither explains the inhibitory interference in antecedent-match conditions nor the difference in effect sizes in antecedent-match vs. antecedent-mismatch conditions.

In order to account for our results and the diverse patterns in the literature, we have introduced two concepts as an extension of the standard cue-based retrieval model. The *prominence principle* implements the idea that a perfectly matching or otherwise highly activated antecedent is only marginally affected by similarity-based interference from comparably poorly matching distractors. This explains the discrepancy between Experiments 1 and 2 (absence of an effect in antecedent-match conditions in Experiment 1 vs. an inhibitory interference effect in Experiment 2). With the concept of *cue confusion*, we proposed that the retrieval cues can be associated with several features of memory items and that the strength of these associations depends on experience with a specific linguistic context. For special cases, this can cause similarity-based interference between items that do not match the same retrieval cues. We argued that *ziji* is such a special case, which would explain the observed inhibitory interference in antecedent-mismatch conditions of Experiment 1.

In the following, we compare the predictions of the extended ACT-R model with the literature on reflexives. Prominence predicts that interference in antecedent-match conditions is generally low compared to antecedent-mismatch conditions but increases as a function of distractor activation. If we assume that distractor position (grammatical role and discourse topicality) affects its base-level activation in memory, the literature summary in Table 1 seems to conform with these predictions: Among the studies which tested both antecedent-match and antecedent-mismatch conditions, about 75% report an interference effect (including marginal effects) in antecedent-mismatch conditions while only 50% of the studies found an effect in antecedent-match conditions. All studies that did report an effect in antecedent-match conditions had the distractor either in subject position (Badecker and Straub, 2002; Chen et al., 2012; Patil, Vasishth, and Lewis, “Retrieval interference in syntactic processing: The case of reflexive binding in English,” unpublished manuscript), in topicalized subject position^15^ (Felser et al., 2009; Cunnings and Felser, 2013; Clackson and Heyer, 2014), or had multiple distractors (Experiment 2 reported here). On the other hand, only half of the studies reporting no interference effect in antecedent-match conditions had the distractor in subject position. Obviously, not all studies that have the distractor in subject position report an effect, but the literature review suggests that subject position increases the probability of finding one. For the absence of an antecedent-match interference effect in our Experiment 1, there might be a specific reason: Dillon et al. (2015) have shown that items within restrictive relative clauses cause more interference as compared to items in appositive relative clauses. They attribute this difference to the idea that, in contrast to restrictive relative clauses, appositive relative clauses constitute a speech act separate from the one of the main utterance (Potts, 2005; Arnold, 2007). More generally, their results suggest that the embedding environment containing a distractor influences the strength of interference caused by this distractor. In terms of ACT-R, one might think of this as different base-level activations as a function of the type of embedding environment. It might be possible that the interposed adverbial structures which contain the distractor in our materials belong to those embedding environments which cause a relatively low degree of interference. This seems a plausible assumption since in our materials, the adverbial clause can simply be ignored by the parser without affecting the grammaticality or plausibility of the whole sentence.

For antecedent-mismatch conditions, cue confusion predicts stronger inhibition the higher the crossed association between cues and features is assumed to be, that is, in contexts with frequently co-occurring cue combinations. However, note that cue confusion is compatible with both facilitatory and inhibitory effects, and even with the absence of an effect, as all this is part of the effect continuum that is illustrated in Figure 2. This raises the concern of how to determine a sensible confusion level in each case, since a model allowing arbitrary predictions is not useful. Currently, the model prediction can only be treated as a predicted difference between two conditions in one or the other direction along the effect continuum. In other words, a prediction should be stated in terms of whether the antecedent-mismatch interference effect of one dependency tends more toward inhibition or toward facilitation in comparison to another dependency like, e.g., English reflexives. In the reasoning we apply here, we refer to English reflexives as a baseline with zero cue confusion and spot special cases where a different feature-co-occurrence rate can be assumed that would motivate a higher confusion level. We have argued that inhibitory interference was observed in antecedent-mismatch conditions in our Experiment 1 because *ziji* is a special case in the sense that the feature combination {+*c-com*, +*animate*} is constant compared to the variable combinations in the different forms of English reflexives. The same logic with respect to {+*c-com*, +*plural*} would apply to reciprocals. In the literature there is one study by Kush and Phillips (2014) that tested the Hindi equivalent of the reciprocal *each other* and indeed found the predicted inhibitory interference in antecedent-mismatch conditions.

Although the *post-hoc* nature of our proposals here is an important limitation that needs to be addressed with new empirical tests, theory development necessarily is data-driven, and the existing data suggest that our proposal constitutes one possible explanation. Indeed, currently it is the only computational account of the patterns of findings discussed here. In order to empirically test the predictions of cue confusion, it is necessary to experimentally manipulate feature-co-occurrence within a minimal pair. A potential experiment could use stimuli like in Example (4) to compare the interference effect in antecedent-mismatch conditions for *themselves* and *each other*. Cue confusion predicts a smaller facilitation or even an inhibition for *each other*. Furthermore, it should be possible to derive a numerical metric of cue confusion for a range of dependencies by computing co-occurrence frequencies in a treebank that contains dependency information as well as information about retrieval relevant features such as gender, number, and animacy.

(4)  a. **Reflexive; distractor-match**
            The *nurse* who cared for the *children* had pricked *themselves* …
      b. **Reflexive; distractor-mismatch**
            The *nurse* who cared for the *child* had pricked *themselves* …
      c. **Reciprocal; distractor-match**
            The *nurse* who cared for the *children* had pricked *each other* …
      d. **Reciprocal; distractor-mismatch**
            The *nurse* who cared for the *child* had pricked *each other* …

A more thorough test of the extended model's predictions will be presented in a forthcoming publication (Engelmann et al., “Confusability of retrieval cues in dependency resolution: A computational model,” manuscript in preparation) that includes quantitative simulations of a range of previous studies on reflexive processing and subject-verb dependencies.

As a rather speculative point we want to add that the cue confusion level of a certain dependency might not only be influenced by feature-co-occurrence but also by task demands and individual differences. If cue-feature associations are subject to an adaptive learning process, they might also be affected by resource-preserving strategies. An example where strategic adaptation of comprehension processes has been found are relative clause attachment ambiguities. Swets et al. (2008) and Logačev and Vasishth (2015) have found that processing effort in ambiguity resolution was adapted to the type of comprehension questions. Also, effects of individual differences in working memory span have been found by Traxler (2007) and von der Malsburg and Vasishth (2012) for the processing of attachment ambiguities. If analogously to task- and resource-related underspecification in attachment ambiguities, cue-feature associations are affected by resource-preserving strategies in the sense of *good-enough processing* (Ferreira et al., 2002), we would expect that low-span readers tend to have greater cue confusion and, thus, exhibit interference effects further toward inhibition in the continuum than high-span readers. The marginal inhibitory effect for low-span readers in antecedent-mismatch conditions of Experiment 2 by Cunnings and Felser (2013) would fit with this expectation. However, more experimental data is needed in order to evaluate effects of individual differences and task-demands on cue-feature associations.

## 6. Conclusion

We have presented experimental evidence that is incompatible with structure-based accounts of reflexive processing and also inconsistent with the original cue-based ACT-R model of sentence processing. In order to account for the observed pattern, we have proposed to add two new principles, prominence and cue confusion, to the ACT-R model. This extension to the ACT-R model is not only able to explain the pattern observed in the data presented in this article, but can also account for a range of previously unexplained patterns reported in the literature on reflexive processing. Naturally, this proposal needs to be evaluated with novel experimental data.

### Conflict of interest statement

The authors declare that the research was conducted in the absence of any commercial or financial relationships that could be construed as a potential conflict of interest.

## Footnotes

^1^Note that the different models of content-addressable memory differ with respect to their assumptions about the exact nature of similarity-based retrieval interference. While the model proposed by Anderson et al. (2004) predicts similarity-based retrieval interference to be observed in retrieval probabilities as well as in retrieval latencies, the model proposed by McElree (2000) predicts that similarity-based retrieval interference only affects retrieval probabilities and not retrieval latencies. In this article, we will focus on cue-based retrieval in the sense of Anderson et al. (2004).

^2^It should be noted that cueing for a c-command feature is a simplification since it actually is a tree-configurational relation between items. There is no straightforward way to attribute a feature like that in an incremental parsing mechanism in content-addressable memory. In this paper, we do not provide a detailed account of how the attribution of a c-command feature could be implemented. As an example, Patil, Vasishth, and Lewis, “Retrieval interference in syntactic processing: The case of reflexive binding in English” (unpublished manuscript) in their ACT-R model for English reflexives approximated a c-command relation by cueing for a subject in the local clause. For a discussion of possible ways to encode tree-configurational information such as c-command in content-addressable memory see Alcocer and Phillips, “Using relational syntactic constraints in content-addressable memory architectures for sentence processing” (unpublished manuscript).

^3^King et al. (2012) report different results in their CUNY 2012 abstract and their final conference poster. We refer here to the results presented on the poster.

^4^There are some exceptions under which the animacy constraint can be violated, see (Tang, 1989; Pan, 1995) for a discussion. Crucially for our experimental design, in the syntactic literature, there is no example of non-emphatic, mono-morphemic *ziji* in argument position bound by a clearly inanimate NP.

^5^The c-command constraint might be violated in case of animate sub-commanding antecedents (Tang, 1989; Xue et al., 1994; Pollard and Xue, 1998), psychological verbs (Huang and Tang, 1991), passives and *ba*-constructions (Yu, 1992, but cf. Cole and Wang 1996), and in case of cataphoric binding by the subject of a matrix clause that is preceded by an adjunct clause containing *ziji* (Huang and Liu, 2001). Moreover, *ziji* can refer to the speaker of the utterance (Li, 1991), the addressee, or even a third person salient in the discourse (Pan, 2000).

^6^In RPD, the effect predicted by the linear-mixed model is also an *inhibitory* one, although the opposite pattern is present in the raw means (cf. Table 3 This discrepancy is driven by a few very long (i.e., >6000 ms) regression-path durations in the antecedent-mismatch/distractor-mismatch condition of one particular item. Because of the concave nature of the log-function, the log-transformation of the data reduces the impact of these extremely high values. As all of these extreme values stem from the same experimental condition, the difference in means of the log-transformed RPDs even switches the sign in antecedent-mismatch conditions (log-transformed means in antecedent-mismatch conditions: distractor-match = 5.85 log-ms; distractor-mismatch = 5.80 log-ms). This explains why the linear-mixed model estimates an inhibitory rather than a facilitatory interference effect. Removing the item which caused the extreme values yields similar results as log-transforming the data, i.e., the sign of the interference effect also switches from negative to positive (raw means in antecedent-mismatch conditions with the item causing extremely long RPDs being removed: distractor-match = 476 ms; distractor-mismatch = 469 ms).

^7^This *t*-value was approximated by using a normal distribution.

^8^Originally, we had 48 items, but 12 of these were excluded based on low acceptability judgments of native speakers.

^9^In response accuracies the proportion of correctly answered yes-questions was strikingly higher than the proportion of correctly answered no-questions. We can exclude the possibility that this pattern can be explained by a general tendency of the participants to answer “yes” since no such difference was observed in filler sentences. We also excluded the hypothesis that this pattern might be related to the difficult nature of the dual-task paradigm by running a follow-up eye-tracking experiment (*N* = 14) with the same experimental set-up but without memory load that yielded a similar response pattern. As the pre-test on the materials had shown that native speakers indeed do the correct binding of the reflexive, we hypothesized that the response pattern was intrinsically related to the nature of the comprehension questions rather than to the experimental sentences themselves. We therefore ran another experiment (*N* = 52) in which the experimental and filler sentences appeared on the computer screen together with the respective comprehension question. Again, we observed a similar response pattern as in the online experiments. We thus conclude that the observed tendency to answer “yes” on the experimental comprehension questions reflects an offline effect, i.e., an effect which occurs at the moment when participants meta-linguistically think about how to answer the question, rather than an effect of online reflexive binding.

^10^The effect of experimental session is not of theoretical interest to our research question, therefore it is not presented in the results tables and will not be discussed further.

^11^This *t*-value was approximated by using a normal distribution.

^12^Note that, for the case of multiple distractors, the original model, too, predicts increased interference. This, however, only explains the difference in effect size between Experiment 1 and 2, but neither the discrepancy between antecedent-match and antecedent-mismatch conditions in Experiment 1 nor the differences between other experiments that did not use multiple distractors.

^13^This is assuming that the pre-critical effects in Experiments 1 and 2 are due to difficulty with an inanimate subject, as discussed above, rather than reflecting an early application of binding during the parafoveal preview of the reflexive.

^14^It should be noted that the (marginal) facilitatory interference in antecedent-match conditions reported by three studies presented in Table 1 (Sturt, 2003; Cunnings and Felser, 2013) is compatible with the ACT-R model although this may not be intuitively obvious. An exceptionally highly activated distractor (in all three of these experiments, the distractor is a discourse prominent subject) can lead to facilitatory interference (see Engelmann et al., 2015, and our forthcoming publication Engelmann, Jäger, and Vasishth, “Confusability of retrieval cues in dependency resolution: A computational model,” manuscript in preparation).

^15^With distractors in “topicalized subject position” we here refer to distractor nouns in subject positions which appear as the current discourse topic in the test sentence because they were introduced in a preceding context sentence.

## Appendix

**Table A1:** **Pretest of Experiment 2: Classification of the participants' answers in the “incorrect” trials by experimental condition**.

**Chosen antecedent of ziji**	**Condition**			
	**Local**	**Non-local**		
Local inanimate subj.	*n.a.*	15 (1.56%)		*Sentence-internal antecedent*
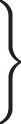
Non-local inanimate subj.	40 (4.17%)	*n.a.*		
Cataphoric binding	40 (4.17%)	14 (1.46%)		
Speaker	11 (1.15%)	4 (0.42%)		*Sentence-external antecedent*
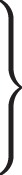
Context	27 (2.81%)	7 (0.73%)		
Author of media noun	19 (1.98%)	0		
Recipient of media noun	2 (0.21%)	0		

Percentages refer to the total number of trials including the correct trials.
